# Cognitive constraints and lexicogrammatical variability in ASD: from diagnostic discriminators to intervention strategies

**DOI:** 10.3389/fnhum.2025.1606701

**Published:** 2025-08-01

**Authors:** Sumi Kato, Kazuaki Hanawa

**Affiliations:** ^1^Department of Neuropsychiatry, Graduate School of Medicine, Hirosaki University, Hirosaki, Japan; ^2^Faculty of Management and Law, Aomori Chuo Gakuin University, Aomori, Japan; ^3^Natural Language Processing Lab, Graduate School of Information Sciences, Tohoku University, Sendai, Japan

**Keywords:** autism spectrum disorder (ASD), natural language processing (NLP), machine learning, diagnostic assessment, corpus, lexicogrammatical discriminator, systemic functional linguistics (SFL)

## Abstract

**Introduction:**

This study examines whether specific lexicogrammatical features can reliably differentiate individuals with autism spectrum disorder (ASD) from non-ASD individuals. Classification models using logistic regression and deep neural networks (DNN) demonstrated high performance—80% accuracy, 82% precision, 73% sensitivity, and 87% specificity. To clarify which linguistic variables contribute to this differentiation, the analysis focused on identifying key syntactic features associated with ASD-specific patterns of lexicogrammatical choices.

**Methods:**

This study used the Tag Linear Model, developed in prior work, which enables identification of specific lexicogrammatical discriminators. Although DNN models achieved higher predictive accuracy, their internal processes were not interpretable. To identify statistically significant features, we applied a logistic regression with 10,000 bootstrap iterations; *p*-values derived from this procedure indicated the statistical significance of each feature. The linear model thus provided transparent evidence of differences in lexicogrammatical features between ASD and non-ASD individuals.

**Results:**

Of the 135 lexicogrammatical items analyzed, 46 were identified asstatistically significant discriminators (*p* < 0.05) between ASD and non-ASD speakers. From these 46 discriminators, 20 showing variation at the clause and phrase level were selected for detailed analysis. These were grouped into seven cognitive-functional domains implicated in ASD, including working memory, inferencing, joint attention, and mental space construction.

**Discussion:**

These findings suggest that syntactic variation in ASD reflects underlying domain-specific cognitive constraints. Linking lexicogrammatical features to cognitive-functional domains provides a linguistically grounded perspective on the neurocognitive profiles of ASD and informs future diagnostic and intervention approaches.

## 1 Introduction

Autism spectrum disorder (ASD) is a neurodevelopmental condition characterized by persistent difficulties in social communication and interactions across various situations. Alongside this, individuals with ASD exhibit repetitive and restricted patterns of behavior, activities, or interests (American Psychiatric Association, 2013). The primary symptom revolves around challenges in social communication, primarily manifesting as pragmatic impairment (Perkins and Firth, 1991; Martin and McDonald, 2003). Pragmatic impairment is characterized by specific difficulties in language comprehension and expression at the pragmatic level, which pertains to the effective use of language in social contexts. This includes challenges in adapting language formality based on the situation, interpreting non-literal language (such as idioms, metaphors, irony, and sarcasm), and understanding the pragmatic aspects of language that affect interpersonal interactions. It refers to struggles with these pragmatic aspects of language, rather than with the basic structural or grammatical components.

There is a widespread consensus among researchers in the clinical field that pragmatic impairment should be examined comprehensively, incorporating multiple factors like language, nonverbal aspects, and cognition. Previous studies have provided insights into the potential factors contributing to pragmatic impairment, indicating that it may arise from neurological, cognitive, symbolic, and/or sensorimotor dysfunctions (Perkins, 2010; Martin and McDonald, 2003; Scobbie, 2005; Murdoch, 1990). Perkins (2010) outlines four key domains of pragmatics, namely: Semiotic, which encompasses language aspects (phonology, prosody, morphology, syntax, semantics, and discourse) and nonverbal elements (gestures, gaze, facial expressions, and posture); Cognitive, which involves processes like inference, theory of mind, executive function, memory, along with emotions and attitudes; Motor, which concerns physical aspects of communication (use of the vocal tract, hands, arms, face, eyes, and body); and Sensory, which focuses on hearing and vision for understanding and conveying information. Perkins’ classification prioritizes factors contributing to pragmatic impairment, framing cognitive dysfunction as the primary cause, with linguistic and sensorimotor factors deemed secondary.

Clinicians have observed individuals with ASD who possess reasonably good language skills but struggle with effective communication. This has led them to recognize the vital role that cognitive functions, such as inferential reasoning, executive function, and memory, play in interpersonal interactions. Consequently, the clinical field has argued for a close association between cognition and pragmatic impairment (Perkins, 2010). As a result, neurology-based research has become a major focus of studies of pragmatic impairment (Martin and McDonald, 2003).

Previous studies regarding concrete linguistic phenomena of ASD with a cognitive perspective explored single grammatical areas such as modality (Perkins and Firth, 1991; Nuyts and De Roeck, 1997; Tager-Flusberg, 1997; Kato, 2021; Perkins, 1983), relative clauses (Durrleman and Delage, 2016; Durrleman et al., 2016), and syntax (Ambridge et al., 2020; Eigsti et al., 2007; Paul and Norbury, 2012; Park et al., 2012; Terzi et al., 2016; Martzoukou et al., 2017; Durrleman et al., 2015).

These studies link linguistic phenomena to cognitive dysfunction but focus narrowly on specific grammatical aspects, leaving the broader impairment uncharted. A comprehensive mapping of pragmatic impairment is needed to identify and analyze linguistic and pragmatic disorders across grammatical domains, yet such a systematic approach remains unexplored.

A comprehensive approach to examining the relationship between language behavior and cognition in ASD involves utilizing spoken language corpora. Previous studies have developed corpora (Nadig and Bang, 2015; Kuijper and Hendriks, 2017; Parish-Morris et al., 2016), but these primarily consist of raw data without detailed linguistic annotation.

For Japanese-speaking individuals with ASD, Sakishita et al. (2020) and Kato et al. (2022) constructed corpora specifically for their research. Sakishita et al.’s corpus, which includes 17 types of phonetic annotations, was analyzed in conjunction with the Autism Diagnostic Observation Schedule, Second Edition (ADOS-2) scores. Kato et al. (2022) developed a more comprehensive corpus focused on syntax and lexicogrammar, based on systemic functional linguistics (SFL). Their annotation scheme includes 159 items derived from the ADOS-2 interview transcripts and story narratives, offering a detailed analysis of lexicogrammatical choices. The corpus comprises 1,187 audiotaped tasks performed by 186 individuals with ASD and 106 non-ASD subjects, encompassing approximately 1.07 million morphemes. This focus on lexicogrammar is crucial, as pragmatic impairment in ASD often manifests through atypical lexical selections and processing difficulties, making it a key indicator of pragmatic impairment.

In the framework of SFL, lexicogrammar refers to the integrated system of vocabulary and grammar, viewed not as separate domains but as two ends of a single continuum. Rather than treating lexis and syntax as distinct levels of analysis, SFL conceptualizes lexicogrammar as the stratum where grammatical structures and lexical choices jointly realize meaning. This perspective enables the analysis of language as a resource for enacting social and cognitive functions in context.

Despite extensive research on pragmatic impairment in ASD (Locke, 1997; Perkins et al., 2006), no prior study has comprehensively analyzed the lexicogrammar of spoken language in ASD. Kato et al. (2022) corpus includes lexicogrammar annotations. Rather than solely identifying pragmatic impairment, this corpus aims to pinpoint ASD-specific lexicogrammatical choices that could serve as diagnostic discriminators.

Building on this framework, Kato et al. (2024) applied machine learning to analyze lexicogrammatical choices in interview and story-recounting texts from 64 ASD and 71 non-ASD individuals (aged 14 and above) to assess differentiation feasibility. Their goal was to develop a Natural Language Processing-based diagnostic tool for ASD, validating lexicogrammatical analysis for assessment. They tested the hypothesis that neurocognitive abnormalities in ASD manifest as distinct linguistic patterns, differentiating ASD individuals from non-ASD through deviations in speech that reflect underlying neurocognitive traits.

Among the most commonly used diagnostic tools for ASD, the ADOS-2 and the autism diagnostic interview-revised (ADI-R) are recommended for combined use due to their high diagnostic validity (Falkmer et al., 2013). The ADOS-2, a semi-structured behavioral assessment, is considered the gold standard for ASD diagnosis (Molloy et al., 2011; de Bildt et al., 2011), while the ADI-R is a caregiver interview providing developmental history and current functioning. However, concerns exist regarding ADOS-2’s versatility, particularly for adults (Adamou et al., 2021; Conner et al., 2019). A major limitation is its difficulty in distinguishing ASD from other neurodevelopmental and psychiatric conditions, such as ADHD (Barlati et al., 2019; De Crescenzo et al., 2019), schizophrenia, anxiety disorders, mood disorders, and avoidant personality disorder (Bresnahan et al., 2009; Luciano et al., 2014; Leyfer et al., 2006). The overlap of symptoms across these conditions (Bastiaansen et al., 2011; de Bildt et al., 2016) further complicates differential diagnosis, as does the heterogeneity within ASD itself. Additionally, masking behaviors, compensation strategies, and learned camouflaging (Gould and Ashton-Smith, 2011; Hull et al., 2017; Lai and Baron-Cohen, 2015) can obscure impairments and contribute to misdiagnosis. Adult diagnosis is further complicated by the frequent absence of caregiver developmental reports and the unreliability of self-insight (Frith, 2003; Berthoz and Hill, 2005).

These results demonstrated the potential of lexicogrammatical analysis as a diagnostic tool. Building on this foundation, the current study seeks to determine which specific lexicogrammatical features serve as reliable discriminators between ASD and non-ASD individuals.

While Kato et al. (2024) demonstrated that neurodevelopmental disorders can be distinguished through lexicogrammatical choices, their study did not pinpoint the specific linguistic features driving this classification. Despite their text + tag DNN model achieving high accuracy (80%), precision (82%), sensitivity (73%), and specificity (87%) for interview texts, the exact lexicogrammatical markers remained unclear. This study builds on previous work by systematically identifying and analyzing the key linguistic features differentiating ASD from non-ASD individuals.

Beyond identifying these features, this study also explores the cognitive mechanisms underlying them. Assuming that language reflects cognitive processing, examining the link between lexicogrammatical choices and cognitive functions provides insights into ASD-specific patterns and pragmatic impairment. In sum, the study connects lexicogrammatical tendencies to neurocognitive traits that shape distinct patterns of language use in ASD. Furthermore, by situating these findings within the broader speech-language continuum—from minimally verbal to highly verbal individuals—this study provides a mechanistic understanding of ASD language variability, supporting the development of interventions tailored to diverse communicative needs across the spectrum.

## 2 Materials and methods

### 2.1 Data

#### 2.1.1 Choice of corpus and participants

This study utilizes the same dataset as Kato et al. (2024), which consists of a spoken language corpus containing speech samples from individuals with and without ASD. The dataset includes 64 individuals diagnosed with ASD (*M* = 18, SD = 3.48) and 71 non-ASD individuals (M = 19, SD = 2.77), all aged 14 and above. The selection of 14 years as the lower age bound was informed by a methodological rationale aimed at minimizing confounds arising from ongoing language development. In line with the position adopted in Kato et al. (2024), we draw on a limited, pro-critical period perspective—not as a theoretical commitment, but as a pragmatic framework for ensuring linguistic maturity in the sample. Specifically, our stance assumes that core morphosyntactic and pragmatic systems are typically stabilized by mid-adolescence, thereby providing a consistent developmental baseline for identifying group-level differences. This view aligns with foundational formulations of the Critical Period Hypothesis in first language acquisition (Lenneberg, 1967; Newport, 1990), while acknowledging the broader spectrum of positions in the literature, including counterevidence from adult learners, neuroplasticity research, and interactionist accounts. Although longitudinal evidence on Japanese is limited, sentence-final particle use and discourse structuring appear to be well established by early adolescence (Clancy, 1985), supporting our rationale for age selection.

##### 2.1.1.1 ASD group

ASD participants were clinically diagnosed using DSM-5 criteria by experienced neurodevelopmental specialists, primarily through the ADOS–2. ADOS-2 assesses social interaction, communication, and repetitive behaviors, with ASD classification based on meeting diagnostic cut-off scores. To ensure a comprehensive evaluation, clinicians also used standardized assessments, including the social responsiveness scale, second edition (SRS-2) for social behavior, WISC-IV (under 16) and WAIS-III/IV (16+) for intelligence, and Vineland-II for adaptive behavior. Autism traits were measured via the autism-spectrum quotient (AQ), while PARS-TR assessed autism-related behaviors through parental reports. Some ASD participants had comorbid conditions, reflecting the high prevalence of coexisting psychiatric and neurodevelopmental disorders in ASD. Rather than isolating ASD from its comorbidities, this study focuses on identifying linguistic markers characteristic of ASD as a whole. Given the rarity of pure ASD cases, an inclusive approach enhances clinical applicability.

##### 2.1.1.2 Non-ASD group

The non-ASD group comprised two subgroups. The first, clinically assessed neurotypical individuals (*N* = 17), underwent the same evaluation as the ASD group but met no psychiatric criteria. Their ADOS-2 scores (3.17 in Module 3, 4.00 in Module 4) confirmed non-spectrum status, with no known neurodevelopmental comorbidities. The second subgroup, non-ASD college students (*N* = 54), was selected based on academic and social adaptability, with ADOS-2 scores (2.01 in Module 3, 4.27 in Module 4) confirming the absence of ASD traits. While they were not assessed for other psychiatric disorders, their successful social functioning strengthens the study’s ability to identify linguistic differences between ASD and non-ASD individuals (see Supplementary Table S1).

#### 2.1.2 Text data

This study analyzes spoken responses from ADOS-2, Modules 3 and 4, which assess verbal fluency, social cognition, and pragmatic language use in verbally fluent individuals. Fluency is defined by the ability to produce complex sentences, logical connectors (e.g., *but, though*), and event descriptions beyond the immediate context, with minor grammatical errors allowed. Module 3, used for adolescents, includes imaginative play, while Module 4, designed for older adolescents and adults, omits this task. Despite this difference, both modules share a consistent structure, enabling comparable data collection across age groups.

#### 2.1.3 Data collection and transcription

Participants with suspected ASD were audio-recorded while completing six to eight tasks in ADOS–2, Modules 3 and 4. These recordings were transcribed and annotated for lexicogrammar, and the resulting texts were stored in the spoken language corpus developed by Kato et al. (2022). The present study used these annotated texts for analysis.

From this corpus, semi-structured interview responses were selected. Topics included personal difficulties, social relationships (friendships, marriage, family), and hypothetical scenarios. The examiner used a conversational approach to encourage natural speech. While both questions and responses were transcribed, analysis focused solely on participants’ speech. No restrictions were placed on text length or word count, ensuring all available speech data was analyzed.

#### 2.1.4 Annotation scheme

Kato et al. (2022) based their annotation scheme on SFL, which views language as a system of context-driven choices. In resource-selection mapping, speakers select from multiple lexicogrammatical options organized in a system network (Martin, 1992). Kato et al. (2024) developed four Japanese system networks: MOOD (clause types, modality), APPRAISAL (evaluative expressions), TRANSITIVITY (experiential representation), and LOGICAL (clausal relationships). These structured networks enable systematic linguistic analysis.

The annotation scheme, detailed in Supplementary Table S2, categorizes lexicogrammatical choices into 15 major categories with 140 tags, offering a comprehensive framework for analysis.

#### 2.1.5 Analysis models

Kato et al. (2024) formulated two diagnostic models: the Tag Linear Model, based on annotated linguistic tags, and a deep learning-based approach that integrates these tags with textual analysis (Tag DNN, Text DNN, and Text+Tag DNN). Among these, the Text+Tag DNN model demonstrated the highest diagnostic effectiveness, achieving 80% accuracy, 82% precision, 73% sensitivity, and 87% specificity.

For the purpose of this study, the Tag Linear Model is the only one that allows direct identification of the discriminator. To ensure comparability across participants, the input to the Tag Linear Model was calculated by normalizing the frequency of each annotated tag by the total number of words produced by each speaker. This procedure allowed us to analyze proportional use of linguistic resources, independent of individual differences in speech quantity.

While deep learning models such as Tag DNN and Text+Tag DNN can capture nonlinear relationships and complex feature interactions—potentially enhancing predictive accuracy—they do not provide a direct explanation of how specific features influence classification. Even though these models were trained on the same annotated features, it cannot be assumed that they relied on the same linguistic cues; deep neural networks often transform and combine features in abstract ways that obscure their specific contributions.

Even so, the transparent evidence provided by the Tag Linear Model supports the existence of lexicogrammatical differences between ASD and non-ASD groups—offering interpretable linguistic insights, even if its statistical performance is slightly lower. This makes the linear model particularly valuable for research focused on identifying specific linguistic markers.

#### 2.1.6 Logistic regression model for identifying discriminators

Due to the small sample size relative to the number of linguistic features, we employed the bootstrap method, which allows for numerical estimation of model parameters.

The bootstrap procedure involved 10,000 iterations, where a random sample was drawn with replacement, and a logistic regression model was trained on each resampled dataset. The coefficients were estimated using numerical optimization techniques. For each coefficient, we computed its mean and variance over the 10,000 iterations. Using these values, we derived the *p*-values to test the null hypothesis that the corresponding coefficient equals zero. These *p*-values indicate the statistical significance of each linguistic feature in distinguishing ASD from non-ASD speech.

## 3 Results

Logistic regression analysis with bootstrap resampling identified 46 lexicogrammatical items as statistically significant discriminators (*p* < 0.05) between ASD and non-ASD speakers. Of these, we selected 20 items that exhibited variation primarily at the clause and phrase level, aligning with the study’s focus on syntactic and lexicogrammatical patterning. These 20 features were categorized according to their relevance to six major cognitive-functional domains implicated in ASD: working memory (WM), inferential ability, mental space management, joint attention (JA), weak central coherence (WCC), self/other differentiation and agency awareness, and restricted and repetitive behaviors (RRB).

**FIGURE 1:**
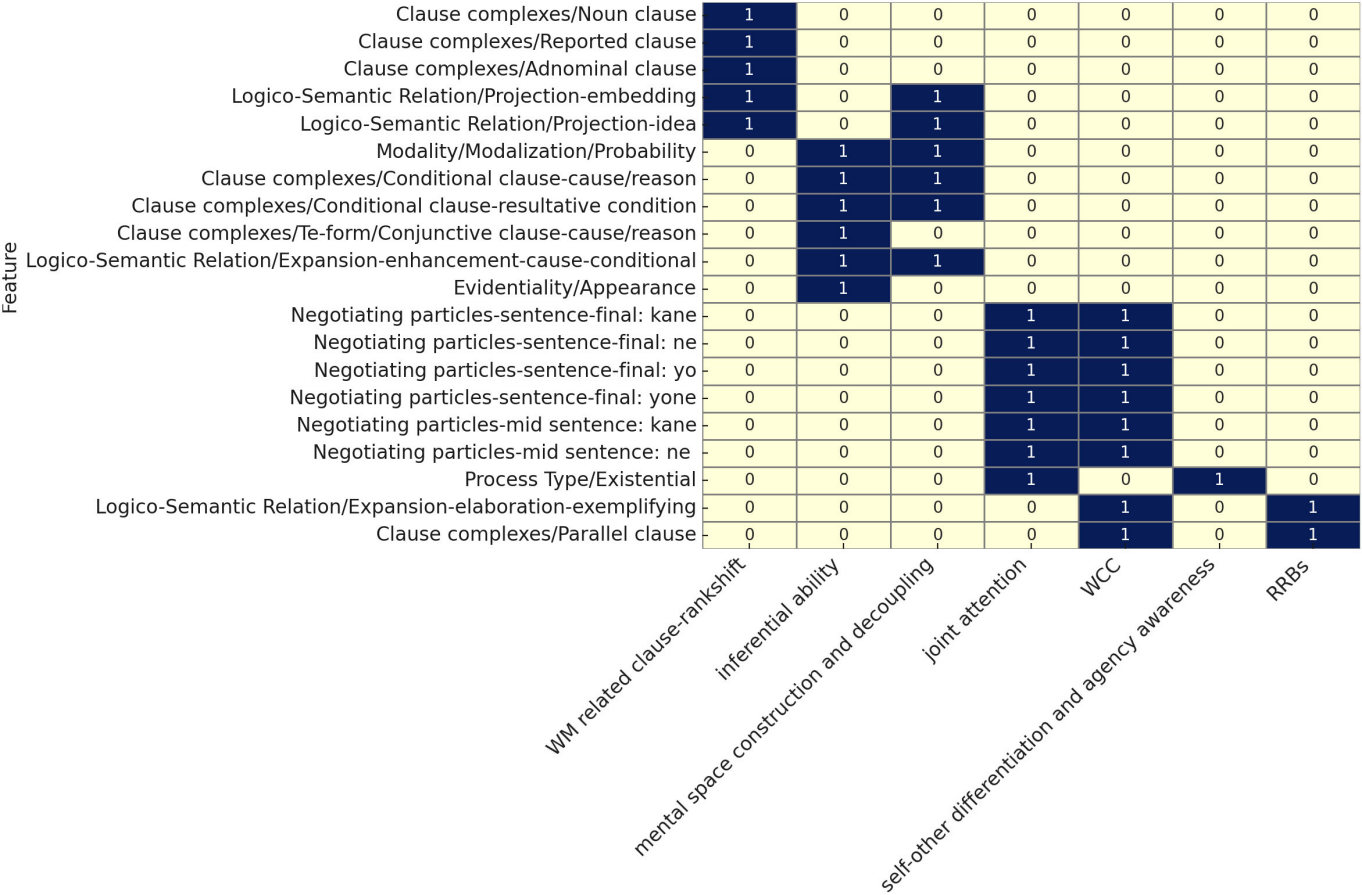
Syntactic variation in ASD: Lexicogrammatical discriminators and cognitive implications.

Table 1 summarizes these discriminators, including their structural type, frequency trends, and *p*-values. Figure 1 provides a functional categorization, mapping each discriminator to the cognitive domain(s) it most clearly reflects. These tables serve as the empirical basis for the interpretive framework developed in the Discussion.

**TABLE 1:** Statistical significance of lexicogrammatical discriminators in differentiating asd from non-asd lexicogrammatical choices.

Lexicogrammar	Mean	SD	*p*-value
Auxiliary verbs-benefactive do (for someone)	−0.0052	0.0018	0.0031
Auxiliary verbs-stative-do (end up/ended up, implying regret/vexation)	−0.0183	0.0075	0.0148
Auxiliary verbs-stative-try (doing/to do something and observe outcome)	−0.0070	0.0032	0.0256
Clause complexes-noun clauses	−0.0503	0.0201	0.0124
Clause complexes-reported clauses	−0.0741	0.0214	0.0005
Clause complexes-adnominal clauses	−0.0512	0.0177	0.0038
Clause complexes/*Te-*form conjunctive clauses (parallel/contrast)	−0.0247	0.0069	0.0004
Clause complexes/*Te-*form/conjunctive clauses-forerunner	−0.0074	0.0030	0.0136
Clause complexes/*Te-*form/conjunctive clauses-cause/reason	−0.0411	0.0103	0.0001
Clause complexes/*Te-*form/conjunctive clauses-attendant circumstance	−0.0185	0.0079	0.0181
Clause complexes/*Te-*form/conjunctive clauses-sequence of actions	−0.0223	0.0084	0.0079
Clause complexes-parallel clauses	0.0808	0.0173	0.0000
Clause complexes-conditional clauses-cause/reason	−0.0381	0.0151	0.0114
Clause complexes-conditional clauses-resultative condition	−0.0227	0.0111	0.0410
Logico-semantic relation/projection-embedding	−0.1052	0.0286	0.0002
Logico-semantic relation/projection-idea	−0.0683	0.0191	0.0003
Logico-semantic relation/expansion-enhancement-manner	−0.0196	0.0083	0.0181
Logico-semantic relation/expansion-enhancement-cause-conditional	−0.1070	0.0250	0.0000
Logico-semantic relation/expansion-extension-additive	−0.0860	0.0221	0.0001
Logico-semantic relation/expansion-elaboration-exemplifying	0.0833	0.0172	0.0000
Process type/existential	0.1336	0.0292	0.0000
Process type/relational-attribute	−0.0849	0.0316	0.0072
Appraisal/attitude/judgment-propriety	−0.0096	0.0042	0.0235
Appraisal/attitude/judgment-veracity	−0.0124	0.0052	0.0163
Appraisal/attitude/affect-satisfaction	−0.0328	0.0049	0.0000
Appraisal/attitude/appreciation-phase-space	−0.0024	0.0011	0.0236
Appraisal/attitude/appreciation-reaction	−0.0533	0.0206	0.0098
Appraisal/graduation/force-intensification	−0.1595	0.0210	0.0000
Appraisal/graduation/force-quantification	−0.0122	0.0057	0.0333
Evidentiality/appearance	−0.0094	0.0041	0.0225
Modality/modalization/modal adjunct/probability	−0.0201	0.0064	0.0016
Modality/modalization/probability	−0.0345	0.0169	0.0410
Modality/modulation/obligation	−0.0042	0.0015	0.0047
Negotiating particles-sentence-final:*kane*	−0.0334	0.0103	0.0012
Negotiating particles-sentence-final:*ne*	−0.1290	0.0281	0.0000
Negotiating particles-sentence-final: *yo*	−0.0329	0.0081	0.0000
Negotiating particles-sentence-final: *yone*	−0.0146	0.0042	0.0005
Negotiating particles-mid sentence:*kane*	−0.0023	0.0009	0.0072
Negotiating particles-mid sentence:*ne*	−0.0363	0.0102	0.0004
Mood/explanatory mood:*kedo*	−0.0347	0.0137	0.0111
Mood/explanatory mood:*ne*	−0.0022	0.0010	0.0199
Mood/explanatory mood:*yo*	−0.0259	0.0063	0.0000
Mood/explanatrory mood:*yone*	−0.0065	0.0022	0.0041
Filler/*unto*	0.0354	0.0135	0.0088
Filler/*kono*	−0.0017	0.0005	0.0021
Onomatopoeia/imitative mimetic words	−0.0126	0.0059	0.0315

### 3.1 WM

ASD participants showed reduced use of syntactically complex, embedded constructions: noun, reported, and adnominal clauses, along with projection-embedding + idea. These structures require hierarchical planning and place demands on verbal WM, suggesting a preference in ASD for flatter, linear syntax.

### 3.2 Inferential ability

Structures involving causal or hypothetical reasoning—conditional clauses (cause/reason, resultative), *te*-form conjunctive clauses-cause/reason, modalization-probability, and evidentiality-appearance—were less frequent in ASD. These forms require integration of background knowledge and contextual inferences, processes often impaired in ASD.

### 3.3 Mental space construction

Mental space builders—projection-idea + embedding, conditional clauses, and modalization-probability—were used less frequently by ASD speakers, indicating difficulty in representing belief states, hypothetical scenarios, or alternative perspectives. This aligns with known deficits in meta-representation.

### 3.4 JA

Negotiating particles (*ne, yo, yone*, *kane*) occurred less often in ASD speech. These forms manage interpersonal alignment and signal shared attention. Their reduced use reflects weaker verbal expression of JA and pragmatic engagement.

### 3.5 WCC

ASD participants showed reduced use of negotiating particles (*ne, yo, yone, kane*) and increased use of parallel clauses and exemplifying elaboration. These patterns suggest a focus on local coherence and reduced sensitivity to discourse-level integration.

### 3.6 Self/other differentiation and agency

ASD participants showed increased use of existential processes (*aru, iru*), which assert presence without indicating agency. This suggests a representational stance favoring observation over interaction, possibly linked to difficulties in agency awareness and self-other distinction.

### 3.7 RRB

Higher use of parallel clauses and exemplifying elaboration in ASD suggests a tendency toward repetitive syntactic patterns, echoing the behavioral rigidity typical of RRB profiles.

In sum, these results identify distinct lexicogrammatical tendencies that differentiate ASD from non-ASD speech, supporting the hypothesis that cognitive constraints shape surface-level syntactic patterns. The following Discussion section interprets these tendencies within a broader cognitive-linguistic framework.

## 4 Discussion

This section builds on the structural patterns identified in the Results, examining how lexicogrammatical discriminators reflect underlying cognitive characteristics associated with ASD. Rather than merely listing group differences, we analyze how reduced or increased use of specific structures—such as embedded clauses, modality, and negotiating particles—corresponds to well-documented cognitive domains, including WM, inferencing, and JA. The 20 discriminators are interpreted in terms of six cognitive-functional domains (Figure 1), allowing us to relate syntactic variation to broader neurocognitive mechanisms. This framework helps to explain how language patterns in ASD are shaped by domain-specific processing constraints, rather than by surface-level variation in expressive style.

### 4.1 Group 1: discriminators of working memory

#### 4.1.1 What is rankshift?

Rankshifted clauses—including noun clauses, reported clauses, and adnominal clauses—along with LSR/projection-embedding and LSR/projection-idea, are significantly less used by individuals with ASD.

Rankshift in SFL refers to the downranking of a clause or phrase so that it functions within a lower-ranked unit, such as a nominal group (Halliday and Matthiessen, 2014). This process enhances meaning-making (semogenesis) by embedding clauses within other structures, enabling more complex and flexible sentence constructions. SFL organizes language hierarchically into clause, group/phrase, word, and morpheme, with rankshift occurring when a higher-ranked unit functions at a lower rank. Examples of rankshift are:

**English**

**(i) nominalization** (clause to noun phrase):

(1) She believes *[that he is honest]*.

Here, *that he is honest* is a clause functioning as the object of the verb ‘believes.’ It is a rankshifted clause serving a nominal role.

**(ii) relative clauses** (clause within noun phrase):

(2) *[The book [that you lent me]]* was fascinating.

The relative clause *that you lent me* modifies the noun *book*, embedding a clause within a noun phrase.

**(iii) adjective phrases within noun phrases:**

(3) *[The man [with the hat]]* smiled.

The prepositional phrase *with the hat* functions as a modifier of *man*, representing a rankshifted phrase embedded within a noun phrase.

**Japanese**

**(i) nominalization using *no* and *koto*:**

(4) [*Kare-ga kuru-no-wo] shitte iru*
  he-NOM come-NMLZ-ACC know PROG
  (I know that he is coming).

The clause *kare-ga kuru* (he is coming) is nominalized using *no*, allowing it to function as the object of the verb *shitte-iru* (know). Interlinear glosses follow the Leipzig Glossing Rules. A full list of grammatical abbreviations used in the glosses is provided in Supplementary Table S3.

**(ii) relative clauses (pre-nominal modification):**

(5) *[[Watashi-ga kinō mita]eiga]*
I-NOM yesterday see-PST movie
(The movie that I watched yesterday).

The clause *watashi-ga kinō mita* (I watched yesterday) modifies the noun, *eiga*, (movie), embedding the clause within the noun phrase.

**(iii) embedded questions:**

(6) *[Kare-ga nani-o itta-ka] oboete-inai*
he-NOM what-ACC say-PST-Q remember-NEG
(I don’t remember what he said).

The clause *kare-ga nani-o itta ka* (what he said) functions as the object of *oboete-inai*, (don’t remember), illustrating a rankshift.

Rankshift is a linguistic mechanism where a higher-rank unit functions at a lower rank within another structure, allowing for complex sentence constructions. For example, a clause can function within a noun group, as seen in relative clauses. Both English and Japanese utilize rankshift, though their grammatical structures and markers differ.

While words and morphemes are part of the rank scale, rankshift primarily involves embedding clauses within groups or phrases rather than focusing on smaller linguistic units. Understanding rankshift helps in analyzing sentence complexity and structural flexibility.

#### 4.1.2 Hybrid approach to complex sentence annotation

Integrating traditional Japanese grammar with SFL provides a comprehensive framework for annotating complex sentences. Traditional Japanese grammar emphasizes syntactic structures, categorizing clauses based on form, such as relative clauses, nominal clauses, and reported clauses. In contrast, SFL focuses on logico-semantic relations (LSR), which describe the logical and semantic relationships between clauses, including elaboration, extension, enhancement, and projection. Combining these approaches allows for an annotation scheme that captures both syntactic structure and semantic function in complex sentences.

One advantage of this hybrid framework is its ability to address overlapping categories. For instance, in the phrase *kino yonda hon*, (the book I read yesterday), traditional grammar classifies the clause as a modifying clause, focusing on its pre-nominal modification. SFL, however, analyzes it as embedding, where a rankshifted clause functions within a nominal group. By integrating both perspectives, this approach ensures a precise representation of both structural and functional aspects of sentence complexity.

Additionally, this combined framework enhances the classification of projection clauses. Traditional grammar identifies nominal clauses as noun-like structures and quotation clauses as reported speech or thought. SFL’s LSR framework categorizes these under projection, divided into projection: idea (thought representation) and projection: locution (speech representation). For example, the sentence *kare-ga kuru-koto-wo shitte-iru*, (I know that he is coming) is a nominal clause in traditional grammar but is analyzed as projection: idea in SFL, representing a mental process. Similarly, *kare-wa asu iku-to itta*, (he said, ‘I will go tomorrow’) is a reported clauses in traditional grammar but falls under projection: locution in SFL due to its reported speech nature. This combined framework ensures a clear distinction between thought and speech projections, enhancing linguistic precision.

Beyond Japanese-specific classifications, integrating SFL facilitates cross-linguistic comparisons. While traditional Japanese grammar focuses on language-specific distinctions, SFL’s functional categories align with universal syntactic and semantic principles, simplifying the comparison of complex sentence structures across languages like Japanese and English.

Practically, this dual-layered annotation scheme offers a flexible and comprehensive analysis. Traditional classifications, such as relative, nominal, and reported clauses, provide clear form-based distinctions, while SFL’s LSR categories, including embedding, projection-idea, and projection-locution, offer function-based classifications. This integration allows researchers to examine Japanese complex sentences from both structural and functional perspectives.

By distinguishing embedded versus ranking elements, projection types, and overlapping categories, this hybrid approach ensures a more accurate and detailed representation of complex syntactic structures. Notably, our annotation scheme incorporates 13 items from the LSR framework, with six identified as discriminators (see Supplementary Table S2). We proceed to analyze specific example sentences to illustrate these concepts.

(i) **A reported clause** is a rankshifted clause that expresses the content of speech, thought, commands, requests, or wishes. In Japanese, it is typically marked by *to* for direct and indirect speech and *yō-ni* for reported thoughts or intentions.

(7) *[Minna-ga watashi-no-koto-wo hen-da] to itteta.*
everyone-NOM I-GEN-thing-ACC strange-COP said-COMP
(Everyone was saying that I was weird).

The clause *minna-ga watashi-no-koto-wo hen-da* (Everyone [was saying] I was weird) is rankshifted and functions as the object of *itte-ta* (was saying).

**(ii) A noun clause** is another type of rankshifted structure that functions as a noun within a sentence. It is commonly formed using formal nouns like *koto* and *no*, allowing the clause to serve as a subject, object, or complement.

(8) *[Sensei-ga yasashii-koto]-ga ureshikatta.*
teacher-NOM kind-NMLZ-NOM happy-PST
(That my teacher was kind made me happy).

The clause *sensei-ga yasashii* (The teacher is kind) is rankshifted and treated as a noun phrase within the larger sentence.

**(iii) An adnominal clause** is a rankshifted clause that functions as a premodifier of a head noun. In SFL, this is analyzed as downranking, where a clause is embedded within a noun phrase, increasing syntactic complexity. The more rankshifted elements a clause contains—such as adnominal clauses—the more structurally intricate it becomes. In Japanese, modifiers follow a regressive pattern, meaning they precede the head noun, whereas in English, relative clauses follow a progressive pattern, appearing after the noun.

(9) English: *The person [I met] yesterday.*

(10) Japanese: *Watashi-ga kinō atta jinbutsu*
I-NOM yesterday meet-PST person
(the person I met yesterday).

In traditional grammar, adnominal clauses correspond to relative clauses, as they add descriptive information to the head noun:

(11) *[[Kono-mae byōin-de hanashita] sensei]-no kotoba-ga zutto kini-natte-iru*.
the.other.day hospital-LOC talk-PST doctor-GEN words-NOM always concern-PROG
(The words of the doctor I spoke to at the hospital the other day have been on my mind).

The clause, *konomae byōin-de hanashita* (who I spoke to at the hospital the other day) modifies *sensei* (*the doctor*).

(12) *[[Densha-de tonari-ni suwatte-ita] hito]-no koe-ga kini-natta*.
train-LOC next.to-LOC sit-PROG.PST person-GEN voice-NOM concern-PST
(The voice of the person who was sitting next to me on the train bothered me).

The embedded clause *densha-de tonari-ni suwatte-ta* (who was sitting next to me on the train) modifies *hito* (person).

These lexicogrammatical structures serve as one of the key discriminators between ASD and non-ASD individuals. We will now probe the cognitive reasoning behind this distinction.

#### 4.1.3 From the cognitive perspective

##### 4.1.3.1 Rankshift and WM constraints

Rankshift occurs when a higher-ranked grammatical unit, such as a clause, is embedded within a lower-ranked unit, like a noun phrase. This increases sentence complexity and demands greater WM resources for processing and production. For example, a simple sentence like *The boy is running* has minimal syntactic dependencies, whereas *The boy who is running is my friend* introduces an embedded relative clause (*who is running*), requiring additional WM capacity.

Individuals with ASD frequently experience WM deficits, particularly in tasks involving language and complex syntax (Adams and Gathercole, 2000, Bentea et al., 2016, Montgomery et al., 2008; Poll et al., 2013). Given that rankshifted structures place higher cognitive demands on an individual’s processing system, those with ASD may tend to avoid using such structures to minimize cognitive load. Instead of constructing sentences with embedded clauses, individuals with ASD may prefer using simpler, sequentially structured sentences. For example, rather than saying *The boy who is running is my friend*, an individual with ASD might rephrase it as *The boy is running. He is my friend.* This restructuring reduces the memory load by eliminating syntactic embedding and presenting the information in a more linear format.

##### 4.1.3.2 Empirical evidence on WM and syntactic complexity in ASD

WM limitations are closely linked to syntactic complexity in ASD, with studies showing that WM deficits correlate with difficulties in sentence comprehension and production (Delage and Frauenfelder, 2012; Montgomery et al., 2009; Eigsti, 2009). Syntactic priming research further indicates that individuals with ASD are less responsive to complex syntactic structures after exposure, reinforcing the idea that such constructions pose a significant processing challenge (Hopkins et al., 2016). This aligns with our finding that participants with ASD produced significantly fewer relative, noun, and complement clauses than their non-ASD counterparts (see Table 1). These clause types require hierarchical embedding and are more demanding in terms of WM and syntactic planning, supporting the interpretation that WM constraints shape spontaneous syntactic expression in ASD.

##### 4.1.3.3 Memory retention and lexicogrammatical development

From a WM perspective, ASD individuals often struggle with long-term memory (LTM) retention of lexicogrammatical structures, affecting sentence construction in spontaneous speech. Lexicogrammar develops through imitating adult speech models, which are stored in LTM and form the basis for adult grammar acquisition (Speidel, 1989, 1993; Speidel and Herreshoff, 1989). However, this process is influenced by WM capacity, particularly the phonological loop (Baddeley, 1986, 2007; Adams and Gathercole, 2000; Montgomery et al., 2008), which temporarily stores verbal information before transferring it to LTM. Impairments in phonological memory disrupt this transfer, inhibiting lexicogrammar development (Gathercole and Baddeley, 1990; Montgomery, 1995; Gillam and van Kleeck, 1996).

In ASD, difficulties in phonological memory may interfere with this process, contributing to reduced syntactic complexity in spontaneous language. The phonological loop, crucial for verbal information processing, is affected in ASD (Adams and Gathercole, 2000; Montgomery et al., 2009), acting as a buffer for sentence processing. Weak phonological memory results in reliance on shorter, syntactically simpler utterances to reduce cognitive load. Instead of embedded structures like relative, noun, or complement clauses, ASD individuals favor flat, linear sentence constructions. This aligns with findings that ASD individuals exhibit lower mean length of utterance (MLU) and reduced lexical density, both markers of simplified syntax (Kato, 2024a).

##### 4.1.3.4 Rankshift avoidance and its pragmatic implications

Since rankshift increases syntactic complexity, its avoidance in ASD may also reflect differences in inferential communication and social cognition. Modifiers and embedded structures serve a pragmatic function, aiding shared understanding. However, ASD individuals often show reduced communicative intent, contributing to their less frequent use of embedded structures. As relative clauses add descriptive content and noun clauses elaborate cognitive states, their avoidance may indicate a preference for direct, less inferential communication (Durrleman et al., 2018).

##### 4.1.3.5 Rankshift and WM constraints

Long-term impact on linguistic development is evident, as these WM limitations persist with age, making the acquisition of lexicogrammar inadequate in its complexity and sophistication (Eigsti, 2009; Montgomery et al., 2009; Delage and Frauenfelder, 2012). As a result, individuals with ASD struggle to acquire complex syntactic structures, leading to a preference for simpler grammatical constructions. The persistence of limited syntactic complexity reflects the long-term impact of WM deficits on linguistic development in ASD. Even in adolescents and adults with ASD, lower MLU values and reduced syntactic variation remain apparent, suggesting that these limitations are not simply developmental delays but rather enduring cognitive constraints. As demonstrated by our findings, the persistence of reduced syntactic complexity with age underscores the need for explicit intervention strategies targeting both WM and syntactic development.

In sum, the avoidance of rankshifted sentences in ASD stems from WM limitations, which affect both syntactic processing and the long-term retention of lexicogrammar. Since rankshift increases syntactic complexity, it imposes greater cognitive demands that individuals with ASD often struggle with, leading them to rely on simpler structures. These difficulties in acquiring and processing complex syntax are not temporary delays but persistent features of ASD language development.

### 4.2 Group 2: discriminators of inferential ability

#### 4.2.1 Context integration and weak central coherence in inferential ability

Discriminators of inferential ability, including modality-probability, conditional clauses, *te*-form conjunctive clauses-cause/reason, expansion-enhancement-cause-conditional, and evidentiality-appearance, are significantly less used by individuals with ASD, reflecting challenges in inferential reasoning. Two primary factors that hinder inferential ability can be considered: (i) context integration difficulties and (ii) weak central coherence (WCC):

(i) *Context integration difficulties*

Contextual integration is crucial for inferencing, allowing individuals to connect linguistic elements across discourse and apply background knowledge. While ASD individuals do not entirely lack inferential ability, they often struggle with making contextually appropriate inferences (Bodner et al., 2015). Even when they understand individual linguistic components, they may fail to integrate them into a coherent whole, affecting their processing of conditional, implicit causal, probable, and evidential relationships.

ASD individuals with strong verbal skills can generate inferences but often produce contextually inappropriate responses that do not align with broader discourse structures (Bodner et al., 2015). They may activate background knowledge but struggle to apply it within a given context, leading to fragmented reasoning and misinterpretation of inferential cues (McKenzie et al., 2010).

These difficulties are most evident in discourse requiring cohesive information linking. Typically developed (TD) individuals process cause-effect relationships across sentences, whereas ASD individuals interpret statements in isolation, leading to breakdowns in inferential comprehension, especially when information is implicit.

Since context integration deficits occur at the discourse level, they do not manifest in specific lexicogrammatical structures, such as conditionals or evidential markers. Instead, they affect the ability to integrate meaning across discourse, making the resulting deficits hard to demonstrate through isolated examples.

(ii) *WCC and the role of sensory precision*

WCC theory posits that individuals with ASD focus on local details rather than global meaning, leading to fragmented linguistic comprehension (Frith, 1989; Happé and Booth, 2008). Instead of forming broader representations of meaning, they process each linguistic unit independently, making it difficult to integrate elements into a coherent whole, particularly in inferential structures requiring contextual linking.

ASD individuals struggle to integrate prior knowledge with real-time sensory input, making it difficult to establish common knowledge (Hohwy and Palmer, 2014). Their model emphasizes that heightened reliance on sensory precision contributes to challenges in processing implicit social cues, affecting inferential discourse. This can also be explained in terms of causal reasoning, where individuals on the spectrum prioritize immediate perceptual details while neglecting broader contextual cues. As a result, this rigidity impairs their ability to infer others’ intentions, align mental states, and establish common knowledge, all of which are essential for understanding inferential discourse.

The following are examples of lexicogrammatical effects:

**(i) conditional clauses (cause/reason, resultative condition)**

**[1] cause/reason conditionals**

(13) *Kanjō-o hyōgen dekita-kara, hoka-no-hito-ga rikai-shi-yasukunaru.*emotion-ACC express can-COND other-GEN person-NOM understand-do-easily become (If one can express emotions, others will find it easier to understand them).

TD individuals flexibly infer cause-effect relationships, while ASD individuals may struggle when causality relies on pragmatic inference rather than direct linguistic cues. They process clauses separately, focusing on surface-level connections, which makes unstated causal links harder to recognize. This reliance on syntactic clarity creates challenges in processing implied causality.

**[2] resultative conditionals**

(14) *Sukoshi yasume-ba, atama-no naka-no moyamoya-ga kieru ki-ga-suru.*
a.little rest-COND head-GEN inside-GEN haze-NOM disappear feeling-NOM do
(If I rest a little, I feel like the fog in my head will clear up).

TD individuals integrate background knowledge to interpret causal relationships, while ASD individuals rely on rigid interpretations, making it difficult to apply contextual information flexibly (McKenzie et al., 2010). This difficulty in updating reasoning when presented with new information affects their ability to process hypothetical or resultative conditionals effectively.

Since these reasoning difficulties arise from multiple cognitive factors, several mechanisms explain why ASD individuals struggle with conditional clauses. First, inhibitory control deficits hinder their ability to suppress reality-based knowledge when considering hypothetical alternatives (Beck et al., 2009). For instance, when hearing *If I had left earlier, I wouldn’t have been late*, TD individuals recognize the implied alternative timeline, whereas ASD individuals may focus solely on the factual outcome of being late.

Second, reduced cognitive flexibility in ASD leads to rigid application of conditional logic, making it difficult to integrate counterexamples or exceptions (McKenzie et al., 2010). This rigidity impairs processing of conditionals requiring perspective shifts, especially in hypothetical reasoning. Contextual integration deficits further compound these challenges, as ASD individuals struggle to adjust conclusions when new information contradicts prior assumptions (Pijnacker et al., 2009).

**(ii) *te-*form/conjunctive clauses-cause/reason**

The *te-*form links multiple clauses, often indicating an inevitable cause-effect relationship. However, ASD individuals may interpret these clauses as separate events rather than a unified causal sequence.

(15) *jikan-ga naku-te mondai-wo subete toku-koto-wa deki-nakatta.*
time-NOM NEG.exist-CONJ problem-ACC all solve-NMLZ-TOP can-NEG.PST
(I couldn’t solve all the problems because I didn’t have enough time).

While TD speakers infer inevitable cause-effect relationships, ASD individuals may struggle to establish this link, leading to fragmented interpretation. Even with explicit reason/cause markers such as *kara* (because), *node* (since), *tame-ni* (in order to), *sei-de* (due to), or *okage-de* (thanks to), they still face difficulties processing causality. This challenge intensifies when causality is implicit, as in *te-*form conjunctive-cause/reason. Without explicit causal markers, ASD individuals may fail to infer the reason-result relationship automatically, relying instead on overt cues, further demonstrating their difficulty in integrating unstated causal links.

**(iii) modalization-probability**

Modalization-probability expresses likelihood, possibility, or certainty. In Japanese, modal markers like *darō* (probably), *kamoshirenai* (might), *ni-chigainai* (must be), and *hazuda* (should be) indicate different degrees of probability.

Example:

(16) *Kare-wa mō ie-ni kaetta hazuda.*
he-TOP already home-LOC returned MOD COP
(He must have already gone home).

TD speakers infer certainty levels using context and background knowledge, but ASD individuals often process probabilistic expressions rigidly, struggling with: (1) interpreting likelihood; (2) adjusting meaning by context; (3) recognizing implied probability without markers.

For instance, *kamoshirenai* (might/maybe) conveys uncertainty, yet ASD individuals may take it as absolute or dismiss it as vague. This challenge worsens when probability is implied rather than explicitly stated, leading to misinterpretations.

**(iv) evidentiality-appearance**

Evidentiality refers to linguistic markers that indicate the source of information, whether directly observed, inferred, or reported (Teruya, 2007). It is categorized into three main types: hearsay, appearance, and reasoning, as shown below. Among these, appearance-based evidentiality was found to be used significantly less frequently.

**[1] hearsay (reported evidence):** refers to cases where the speaker conveys information obtained from others rather than direct experience. The marker *sōda* (I hear) is commonly used for this function.

(17) *Tenkiyohō-ni yoru-to, ashita-wa hareru sōda.*
forecast-DAT according-COND tomorrow-TOP sunny-HEARSAY
(According to the weather forecast, it will be sunny tomorrow).

In this sentence, the speaker relays information from a source rather than making a direct observation.

**[2] appearance (sensory-based inference):** involves judgments based on visual or sensory perception. The speaker makes an inference from observed sensory input, rather than reporting or reasoning from background knowledge.

(18) *Kono sūpu-wa atsusō-da.*
this soup-TOP hot-APPEAR-COP
(This soup looks hot).

Here, the heat of the soup is inferred from visible cues such as steam or bubbling liquid rather than from direct contact.

**[3] reasoning (cognitive inference):** refers to cases where the speaker makes a conclusion based on logical deduction or contextual knowledge rather than sensory perception. This is commonly expressed in Japanese with *darō* (I guess) or *yōda* (seems).

(19) *Hikōki-wa mō kūkō-ni tsuita-darō.*
airplane-TOP already airport-LOC arrive-MODAL
(The airplane must have landed at the airport).

The speaker does not directly witness the event but infers it based on expected timing and general knowledge of flight schedules.

Among the three types of evidentiality, the reduced use of appearance-based evidentiality in ASD may stem from atypical sensory perception. Some individuals experience hypersensitivity, making them distrust sensory input and avoid markers like *sōda* (looks/seems). Others, with reduced sensitivity, may miss relevant cues, hindering sensory-based inferences. These variations in sensory perception likely contribute to the observed patterns in evidentiality use, leading ASD individuals to rely less on appearance-based evidentiality.

#### 4.2.2 Distinction between modalization-probability and evidentiality-reasoning

Although both probability and reasoning involve uncertainty, they function on fundamentally different principles.

**(i) probability as an internal judgment**: refers to the speaker’s internal assessment of likelihood, based on their own reasoning or inference. It reflects a subjective evaluation rather than an externally sourced fact.

(20) *Hikōki-wa mō kūkō-ni tsuita-hazuda.*
airplane-TOP already airport-LOC arrived MOD COP
(The airplane must have already landed at the airport).

The phrase *hazuda* (must) expresses a high degree of certainty but remains an internal assumption based on contextual knowledge rather than external evidence.

**(ii) evidentiality as an external source of knowledge**: indicates the information source—whether directly observed, inferred from sensory input, or reported by others (Teruya, 2007). Unlike probability, it concerns how the speaker knows something rather than their level of certainty.

(21) *Hikōki-wa mō kūkō-ni tsuita-darō.*
airplane-TOP already airport-LOC arrived MOD
(I guess the airplane has already landed at the airport).

The *darō* (I guess) here functions as both a probability and an evidential marker, implying that the speaker is making an informed guess, likely based on general knowledge rather than direct observation.

In SFL, Kadooka et al. (2016) categorizes evidentiality as a subtype of modality, whereas Teruya (2007) treats it as an independent system related to propositional validity, which aligns with Kamio (1990), who argues that absolute evidence is unattainable in conversation, meaning that all statements rely on some form of inference—whether subjective (probability) or based on external sources (evidentiality).

#### 4.2.3 Impact of comprehension deficits on spoken language

ASD individuals struggle with inferential language due to deficits in common knowledge, social inferencing, sensory processing, and limited exposure to inferential discourse. These challenges affect both comprehension and lexicogrammatical choices.

The discussion so far has focused on input processing, but in ASD, deficits in inferential comprehension also impact language output. Difficulty interpreting implicit meanings and complex relationships leads to a preference for simpler, more explicit structures. Lacking a strong grasp of syntactic complexity and inferential cues, ASD speakers rely on straightforward constructions. In essence, what one cannot infer, one cannot effectively produce.

### 4.3 Group 3: discriminators in mental space construction and decoupling

Discriminators in mental space construction including LSR-projection (idea + embedding), modalization-probability, and conditional clauses, are significantly less used by individuals with ASD, reflecting difficulties in structuring conceptual relationships beyond direct real-world references within the mental space theory framework. Mental space theory is introduced first.

#### 4.3.1 Mental space theory and its role in language understanding

Fauconnier (1994) introduced mental space theory to explain how language constructs cognitive representations beyond direct real-world reference. Instead of assuming that linguistic expressions correspond directly to external reality, mental space theory posits that meaning is structured through mental spaces—temporary cognitive domains that allow individuals to interpret different perspectives, hypothetical scenarios, and belief states. Mental space theory integrates pragmatics (context-based meaning) and semantics (linguistically encoded meaning) to explain how we comprehend language beyond surface-level interpretation.

**FIGURE 2:**
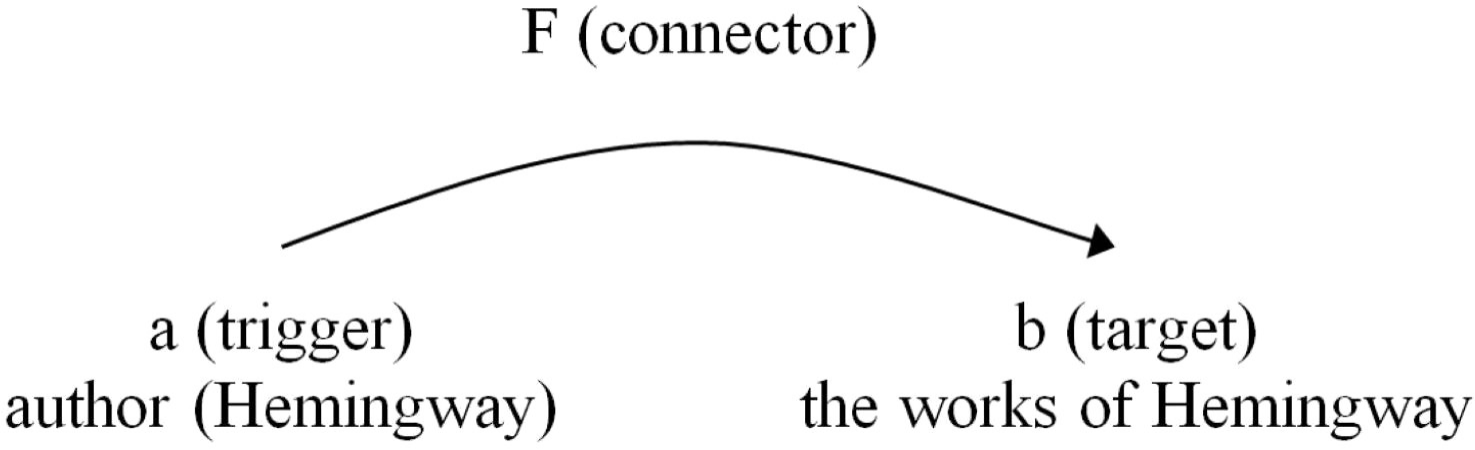
Metonymic mapping of author and literary works.

A classic example is metonymy, where an author’s name represents their literary works:

(22) *I read Hemingway* → *Hemingway* functions as a stand-in for his works, illustrating the Identification (ID) principle, which maps a conceptual link between the trigger (author’s name) and the target (his works) (Figure 2).

While metonymy relies on direct associations, metaphor processing—a well-documented challenge for individuals with ASD (Happé, 1993; Norbury, 2005; Kalandadze et al., 2021)—requires more complex conceptual mapping across mental spaces. Understanding metaphors involves linking distinct conceptual domains, allowing individuals to interpret abstract meanings beyond literal word definitions. For example, interpreting *Knowledge is light*—a metaphor that maps illumination onto understanding—requires mental space construction.

Mental space theory further explains belief spaces, demonstrating how mental spaces allow us to process different perspectives:

(23) *Len believes that the girl with blue eyes has green eyes.*

**FIGURE 3:**
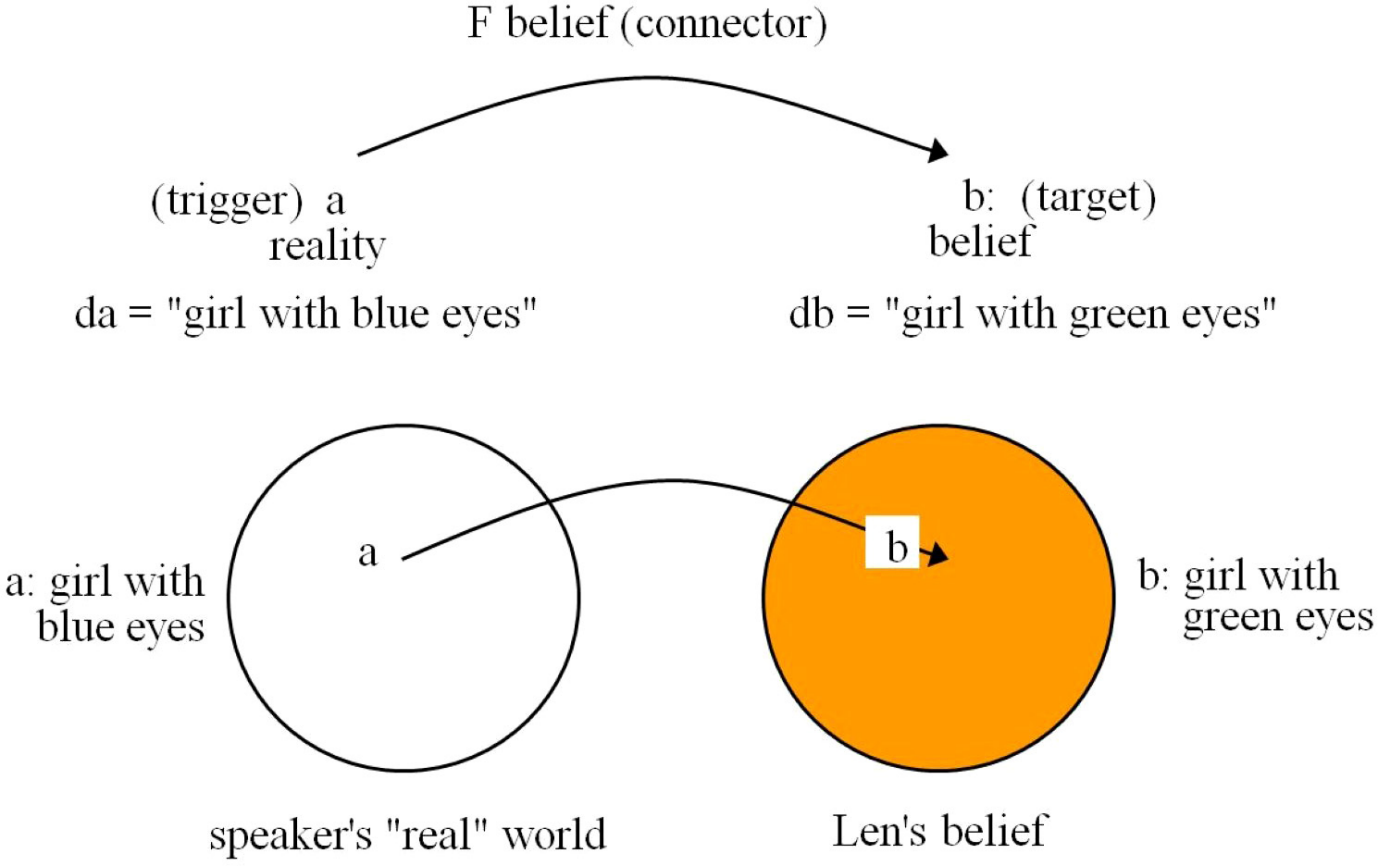
Belief spaces and the ID principle in mental representation (Fauconnier, 1994: 12, 14).

Here, *believes* triggers a shift from the speaker’s own reality to Len’s subjective belief, creating a mental space distinct from objective reality (Figure 3). This showcases how conceptual mappings guide meaning interpretation.

#### 4.3.2 Mental space builders and their role in ASD

In discourse, mental space builders (e.g., *if A, then B*; *Max believes X*; *in Len’s mind*) establish and structure mental spaces, facilitating shifts between different perspectives and realities. These linguistic elements are essential for processing counterfactual reasoning, hypothetical scenarios, and belief attribution.

**FIGURE 4:**
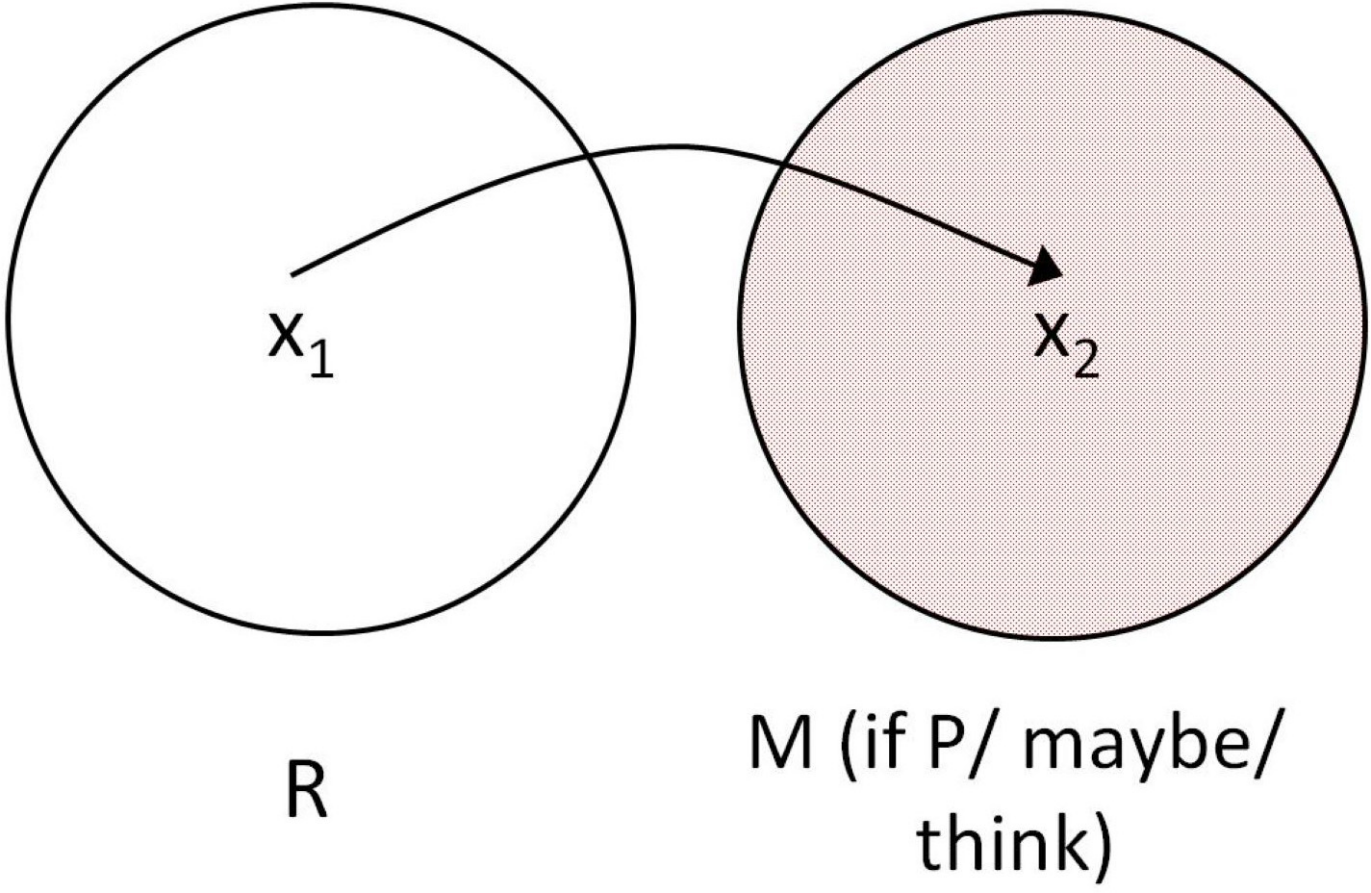
Mental space theory and metaphor processing in ASD.

This study found that individuals with ASD exhibit significantly less frequent use of mental space builders, suggesting difficulty in constructing and managing mental spaces. This may contribute to challenges in understanding hypothetical reasoning, interpreting figurative language, and shifting between different perspectives in discourse (Figure 4).

Individuals with ASD, who tend to process language literally, may struggle to establish such conceptual mappings, leading to difficulty in understanding non-literal expressions. This study identifies the following discriminators in ASD and non-ASD:

**(i) projection-idea;** represents subjective thoughts and beliefs, often marked by *to omou* (I think) in Japanese. This functions as an mental space builder by shifting discourse from an objective statement to the speaker’s internal cognition or belief state.

(24) *Okkina shiren-toka konnan-ga machiuketeiru-to omou-n-desu-yo.*
big ordeal-ETC difficulty-NOM await-PROG-COMP think-NMLZ-COP-FP
(I think there will be big ordeals and difficulties awaiting).

Here, *to-omou* (I think) creates a mental space distinct from objective reality, framing the content as the speaker’s subjective belief rather than an asserted fact.

**(ii) Modalization-probability**; expresses uncertainty, possibility, or hypothetical reasoning, often using kamoshirenai (might) in Japanese. As an mental space builder, it shifts the meaning into a hypothetical space, marking the statement as non-definitive.

(25) *Nige-kireru kamoshirenai-kedo rōka-de hashittara okorareru-shi.*
escape-can MODAL-but hallway-LOC run-COND scold-PASS-CONJ
(I might be able to get away completely, but if I run in the hallway, I will get scolded).

Here, *kamoshirenai* (may/might) creates a mental space of uncertainty, where the event is not confirmed but considered possible. ASD individuals, who often prefer explicit and concrete statements, may struggle with this probabilistic reasoning.

**(iii) Conditional clauses**; establish hierarchical relationships between clauses, structuring causal, adversative, or hypothetical relationships. In mental space theory, these mental space builders introduce dependency between mental spaces, signaling how one event relates to another.

[1] Cause/reason conditional

(26) *Jibun-no kimochi-ga wakaranai-nara, sore-o kotoba-ni suru-no-wa muzukashii.*
self-GEN feeling-NOM understand-NEG-COND it-ACC words-LOC put-NMLZ-TOP difficult
(If you don’t understand your own feelings, putting them into words is difficult).

[2] Resultative Conditional (Causal Relationship: If X Happens, Y Follows)

(27) *Minna-ga waratte kureru-to ureshii-desu.*
everyone-NOM laugh-BEN-COND happy-COP
(I am happy if everybody laughs).

Here, *to* (if) builds a conditional mental space, where the speaker’s emotional response (I am happy) is dependent on the condition (everybody laughs).

The reduced ability to construct mental spaces in ASD may explain challenges in metaphor comprehension, pointing to the role of mental space builders in conceptual thought and discourse navigation. In sum, mental space theory provides a framework for understanding how language constructs meaning beyond direct reference. Reduced mental space builder use in ASD suggests difficulties in mental space construction, contributing to challenges in hypothetical reasoning, belief attribution, and metaphor comprehension. To explain this, we adopt the decoupling perspective.

#### 4.3.3 Interpretation from the perspective of decoupling

Decoupling is the cognitive ability to separate immediate perceptions from alternative representations, supporting imagination, flexible thinking, and abstract reasoning. Leslie (1987) introduced this concept in relation to pretend play, where children distinguish reality from pretense. For example, when a child pretends a banana is a telephone, they must decouple its real function as food from its imagined use, avoiding confusion.

Decoupling is a key mechanism of meta-representation—the ability to form representations of representations (Leslie, 1987). His model views pretend play as an early example of this ability, involving three processes: the *Expression Raiser*, which copies and disconnects a primary representation from reality; the *Manipulator*, which integrates the decoupled representation into a pretend scenario; and the *Interpreter*, which reconnects it to real-world interactions (Leslie and Roth, 1993).

Difficulties in decoupling have been linked to ASD, where individuals struggle with pretend play, flexible thinking, and understanding others’ beliefs, thoughts, and emotions (Leslie and Roth, 1993; Kinoshita, 1992; Frith, 1989). Reduced spontaneous pretend play in ASD suggests decoupling deficits, contributing to social and communication challenges (Wing and Gould, 1979). Beyond pretend play, decoupling is crucial for understanding non-literal language, including metaphors, irony, and figurative expressions. Impairments in this area lead to overly literal interpretations and communication difficulties.

While Leslie’s model has been highly influential, Perner (1988, 1991) argued that pretend play relies on a simpler *as if* representation rather than meta-representation, allowing children to engage in pretense without understanding mental states. Other researchers (Wellman, 1990; Astington, 1996) challenge Leslie’s emphasis on innate cognitive mechanisms, instead emphasizing the role of environmental and cultural factors in cognitive development.

Despite these debates, decoupling remains central to cognitive flexibility, imagination, and social cognition. Its impairment in ASD helps explain difficulties in communication, perspective-taking, and abstract thinking.

#### 4.3.4 Decoupling dysfunction and mental space builders in ASD

Even in ASD individuals with strong linguistic skills, difficulties in symbolic reasoning and communication persist (Leslie and Roth, 1993). The significantly lower use of mental space builders in ASD suggests cognitive differences in:

(i) projection-thinking (belief space): difficulty distinguishing between primary (real) and secondary (imagined) representations.
(ii) Modalization-probability (possibility space): reduced ability to evaluate likelihood and hypothetical scenarios.
(iii) Conditional clauses (hypothetical space): impaired reasoning about counterfactuals and cause-effect relationships.

While ASD individuals retain some decoupling ability, its efficacy varies across the spectrum. Impairments in meta-representational abilities result from a malfunction in the decoupling mechanism (Leslie and Roth, 1993). This dysfunction is an innate cognitive impairment rather than a general developmental issue (Leslie, 1991).

This study, focusing on individuals aged 14 and older, does not address whether cognitive abilities are innate or acquired but examines how decoupling dysfunction affects linguistic expression. The significantly lower use of mental space builders in the ASD group suggests distinct cognitive traits, indicating difficulties in distinguishing reality from mental representations—key to constructing mental spaces, similar to pretend play (Kato, 2025, in press).

A broader theoretical issue arises: if decoupling deficits explain the reduced use of mental space builders, does this suggest mental space construction is not an independent cognitive function but a secondary effect of decoupling ability? This question leads to a fundamental issue regarding mental space theory’s cognitive status and its relationship with decoupling (Kato, 2025, in press).

#### 4.3.5 Reevaluating the cognitive basis of mental space construction

Takubo (1992) views mental space construction as a cognitive construct that actively organizes semantic and pragmatic information in real-time processing. Under this view, mental spaces function as cognitive tools for counterfactual reasoning, belief attribution, and conceptual organization, suggesting mental space construction is an intrinsic cognitive mechanism. In contrast, Sakai (2014) argues that mental space construction is not an independent cognitive function but a symbolic system modeling how language encodes conceptual structures. Mental space construction, in this view, does not govern real-time cognition but serves as a linguistic mapping tool within discourse. Thus, while Takubo sees mental space construction as a core cognitive mechanism, Sakai considers it a framework shaped by other cognitive abilities.

This study aligns with Sakai’s perspective, suggesting that ASD individuals’ reduced use of mental space builders stems from decoupling deficits rather than mental space construction impairments. Decoupling, as described by Leslie (1987), allows individuals to separate real-world knowledge from hypothetical scenarios, a crucial ability for constructing mental spaces (Kato, 2025, in press). However, Fauconnier (1994) did not explore mental space theory’s cognitive basis, leaving open whether mental space construction relies on specialized neural mechanisms or broader cognitive functions. If mental space construction is only a symbolic mapping system, its role in language comprehension must be reconsidered in relation to foundational cognitive abilities.

Future research must clarify whether mental space construction is: (i) an independent cognitive construct or (ii) a system shaped by other cognitive mechanisms. Resolving this distinction has significant implications for understanding language processing differences between TD and atypical populations.

### 4.4 JA discriminator: negotiating particles

#### 4.4.1 The role of negotiating particles in Japanese interpersonal communication

Negotiating particles, found to be used significantly less by ASD individuals, serve as a discriminator in JA. Negotiating particles are essential in Japanese interpersonal communication. Particles like *ne* and *yo* appear at clause endings to facilitate interaction. For example, *…shimasu* (do) becomes an interpersonal statement with *ne* (*…shimasu-ne (yo)*), engaging the listener. English lacks direct equivalents, instead relying on intonation, modal verbs, or discourse markers (*right?*, *you know*, *isn’t it?*). However, these elements do not function as fixed grammatical particles like their Japanese counterparts.

These particles are lexicogrammatical elements that convey the speaker’s attitudinal stance toward a proposition or proposal (Teruya, 2007). They regulate turn-taking, attention management, and stance negotiation in discourse. In particular, *ne* and *yo* frequently serve interpersonal functions. *Ne* is associated with confirmation and shared cognition, fostering social alignment. *Yo* conveys insistence, assertion, or emphasis, marking statements as informative or directive (Nihon-kijutsubunpo-kenkyukai, 2008). *Yone* combines *yo* and *ne*, indicating that the speaker believes their cognition is acceptable to the hearer. *Kane* blends the interrogative *ka* with *ne*, introducing a questioning function while softening inquiries.

Maynard (1997) analyzed 60-min daily conversations among 20 pairs of subjects and found that sentence-ending particles accounted for 35% of all sentence-final expressions, with *ne* and *yo* being the most frequently used. In Japanese socio-cultural contexts, these particles reflect the speaker’s intentions regarding interpersonal distance and relationship-building with the interlocutor.

(i) Ownership of information and negotiating particle selection

The use of *ne*, *yo*, and *yone* is influenced by how the speaker perceives the ownership of information. A reduced use of these particles in spoken Japanese among ASD individuals is considered an indicator of pragmatic impairment, as these structures are essential for engagement and shared cognition. The selection of negotiating particles depends on the relative degree of information ownership between the speaker (S) and hearer (H) (Table 2).

**TABLE 2:** Speaker-Hearer information ownership and negotiating particle selection.

Speaker-hearer information ownership	Preferred particle
S exclusively holds the information; H does not have any	*yo*
H exclusively holds the information; S does not have any	*ne*
S’s amount of information is greater than H’s	*yo* or *yone*
H’s amount of information is greater than S’s	*ne*
S and H have the same amount of information	*ne*

S, speaker; H, hearer.

The information ownership framework demonstrates how these particles mediate epistemic relationships, signaling the speaker’s assumption about what the listener already knows. *Yo* is used when the speaker possesses more information than the listener, but it may be softened with *yone* when the hearer is expected to confirm the statement (Maynard, 1993). *Yo* often signals new information (Kitagawa, 1984), draws the hearer’s attention, or prompts action (Shirakawa, 1992; Izuhara, 2001). Compared to *ne*, which seeks confirmation and attunement, *yo* carries a stronger directive force and asserts the speaker’s viewpoint more forcefully (Imamura, 2011; Tokieda, 1951). While both particles regulate interpersonal interaction, *yo* has a stronger attention-calling function (Izuhara, 2001).

(ii) Pragmatic functions of *ne* from a calling-attention perspective

The particle *ne* plays multiple roles in managing attention and interaction in discourse (Maynard, 1997) and also serves politeness-related functions (Usami, 1997; Table 3).

**TABLE 3:** Pragmatic and politeness functions of the particle *ne* in discourse.

Function	Description	Source
Attention and interaction management		Maynard (1997)
Initiating interaction	Engages the hearer by introducing a topic.	
Conversational bonding	Reinforces solidarity by indicating shared experiences.	
Seeking confirmation	Requests the hearer’s agreement on shared information.	
Politeness-related functions		Usami (1997)
Promoting conversation	Encourages continued interaction and shared cognition.	
Calling for attention	Draws the hearer’s focus to the speaker’s statement.	
Softening utterances	Reduces imposition as a negative politeness marker.	
Confirming content	Makes statements less confrontational.	
Compensating for an utterance	Reinforces politeness through expressions like *desu-ne*.	

#### 4.4.2 Negotiating particles and pragmatic impairment in ASD

In spoken Japanese, *ne* and *yo* are crucial for managing attention and interpersonal alignment. Their role in drawing the hearer’s focus and fostering joint understanding suggests that reduced use among ASD individuals reflects pragmatic impairment. This study links their diminished use to deficits in JA, a key ability in social cognition and communication development.

The cognitive function of *ne* in calling for attention directly connects it to JA, a key mechanism in early social-cognitive development (Mundy et al., 1990). ASD individuals frequently exhibit difficulties in gaze-following, pointing, and object-sharing, which are essential for coordinating social interactions (Leekam, 2005). Since JA is linked to social motivation (Mundy and Sigman, 2006), children who are more socially engaged tend to develop stronger communication skills. However, ASD individuals often lack social motivation, process emotions differently, and exhibit reduced orientation to faces (Dawson et al., 2005; McPartland et al., 2011), limiting their opportunities for language socialization. JA consists of two components; Responding to JA and Initiating JA. Responding to JA and initiating JA are supported by distinct but interrelated neural networks, with initiating JA being more closely tied to social motivation (Schilbach et al., 2010; Schietecatte et al., 2012). Since *ne* and *yo* serve as verbal markers of JA, their reduced use in ASD individuals suggests weak social motivation and impaired initiating JA.

In sum, *ne, yo, yone*, and *kane* serve as key markers of social alignment, information ownership, and attention management. Their reduced use in ASD individuals reflects pragmatic impairment, weak social motivation, and JA deficits. As these particles facilitate interpersonal engagement, their absence suggests broader communication challenges, reflecting cognitive-linguistic difficulties in ASD social interaction (Kato et al., 2022).

### 4.5 Self-other differentiation and agency awareness discriminator: existential process

#### 4.5.1 The focus on existential processes in ASD

ASD individuals use the existential process significantly more, making it a discriminator in self-other differentiation and agency awareness. In SFL, the process system refers to how language represents different types of experiences through the transitivity system. The clause serves as the primary unit for expressing these experiences, and the process system organizes human experiences into six distinct Process types (Table 4; see Supplementary Figure S1).

**TABLE 4:** Process types in the transitivity system of SFL.

Process type	Description	Example
Material processes	Represent physical actions and events.	*She opened the door.*
Mental processes	Express thoughts, feelings, and perceptions.	*He believes the story.*
Relational processes	Indicate states of being, attributes, or identity.	*This book is interesting.*
Behavioral processes	Bridge physical actions and mental states, representing behaviors.	*The baby is crying.*
Verbal processes	Represent acts of saying, reporting, or communicating.	*She said the meeting was at noon.*
Existential processes	Indicate the existence or presence of something.	*There is a book on the table.*

The transitivity system in SFL structures human experiences into linguistic categories, allowing language to not only describe reality but also construct it. By distinguishing between different types of processes, language provides a systematic way of organizing how we perceive actions, cognition, relationships, behaviors, speech, and existence.

This study reveals significant differences in existential process use between ASD and non-ASD individuals, with ASD individuals using existential processes more frequently. Existential processes indicate existence or presence without implying action or emotion, distinguishing between animate and inanimate subjects: *iru* (be) for animate entities and *aru* (be) for inanimate ones.

(28) *Tsukue-no ue-ni hon* (Existent)-ga *aru* (Process: Existential: inanimate)
desk-GEN top-LOC book-NOM exist
(On the desk, there is a book).

(29) *Isu-no ue-ni neko* (Existent)-ga *iru* (Process: Existential: animate)
chair-GEN top-LOC cat-NOM exist
(On the chair, there is a cat).

In Japanese, *iru* (animate) and *aru* (inanimate) express both existence and possession, unlike English, which separates BE-type (existence) and HAVE-type (possession) verbs (Takezawa, 2003). These verbs indicate: (1) inherent ownership; (2) human relationships (e.g., kinship); (3) part-whole relationships (e.g., body parts); (4) spatial relationships (Chappell and McGregor, 1996). While (1)-(3) reflect possession (X has Y), (4) conveys existence (Y is at X), aligning with transitive (possession) and intransitive (existence) structures (Kishimoto, 2003). English *have* can imply existence (We have much snow), showing overlap between HAVE and BE verbs (Yamaguchi, 2003), but in Japanese, *aru* and *iru* are more natural for existence.

Given this distinction, *aru* and *iru* play a central role in existential processes, serving as core BE-type verbs that express existence (*exist, happen, arise*) or circumstance (*sit, stand, grow*), with 74% of existential process expressions in this study involving *aru* and *iru*.

ASD individuals showed significantly higher existential process use, especially for spatial relationships. This section examines differences in existential process use between ASD and non-ASD groups, analyzing them in terms of self-other awareness, JA, and sense of agency.

#### 4.5.2 Ecological self and interpersonal self as binary relations

Neisser (1988) identified 5 types of self-knowledge, with this study focusing on two: (1) ecological self—the most basic self-awareness, emerging from sensory experiences and physical interactions. (2) interpersonal self—self-awareness formed through social interactions and emotional exchanges.

The ecological self reflects how individuals perceive themselves via direct environmental engagement, like navigating a dark room using tactile and auditory sensations. In contrast, the interpersonal self emerges through early social interactions, such as infant-caregiver reciprocity, which forms the basis for understanding social relationships (Honda, 2005).

Infants initially form binary relationships, focusing on either their environment (e.g., object attachment) or people (e.g., bonding with caregivers). By 9–12 months, these relationships merge into a ternary relation, enabling infants to perceive objects within shared social contexts. This marks the emergence of JA, a key milestone in social and cognitive development (Mundy et al., 1990).

#### 4.5.3 Self-other differentiation and existential processes

The significantly higher frequency of existential process constructions in the ASD group, especially those involving inanimate subjects (e.g. *hon-ga aru*, ‘there is a book’; *isu-ga aru*, ‘there is a chair’), reflects not just a stylistic preference but a fundamentally different mode of world construal. Existential processes serve to assert the presence of entities or conditions without specifying agentive or experiential participation. In systemic functional terms, this process type encodes existence rather than action, perception, or mental engagement. The prominence of existential processes in the ASD group suggests a representational stance centered on environmental registration rather than interactional positioning. Unlike mental or material processes that presuppose relational dynamics between participants (e.g., Actor and Goal, Senser and Phenomenon), existential processes foreground isolated states of being. This aligns with the idea that individuals with ASD may remain anchored in what Neisser (1988) terms the *ecological self*—defined by one’s perception of the immediate environment as opposed to intersubjective awareness. Previous studies suggest this ecological orientation persists in ASD beyond infancy, with delays or deficits in the emergence of the interpersonal self (Honda, 2005). The ASD group’s reliance on existential processes reflects this persistence in linguistic form: a grammar of observation rather than engagement. Although Japanese allows for subject omission and implicit agency (Ikegami, 2006), the distributional skew observed in our corpus suggests a cognitive-linguistic divergence not explained by typological norms alone.

#### 4.5.4 JA and the interpersonal self

The transition from ecological to interpersonal self-awareness is typically mediated by the emergence of JA, which allows individuals to coordinate attention with others toward shared referents. This ability supports the development of shared intentional frameworks and the recognition of others as intentional agents. Research has consistently found that JA is impaired in ASD (Mundy et al., 1990; Leekam, 2005), with downstream effects on social cognition and language development. These impairments appear in reduced frequency and complexity of gaze-following, pointing, and protodeclarative gestures—behaviors that scaffold shared meaning. In linguistic terms, the ability to construe another as a co-present participant underlies the selection of animate-subject existential processes (e.g., *neko-ga iru* “There is a cat”). These constructions index not only presence but co-awareness—an acknowledgment of an animate being as a potential object of shared attention. The ASD group’s low usage of such constructions suggests a diminished capacity or motivation to encode this interpersonal alignment. As with JA behaviors, the grammatical data reflect the absence of a ternary relation (self–other–object) and a retreat into simpler dyadic structures (self–object). This interpretation is consistent with neurocognitive findings. Chiu et al. (2008) found reduced activation in the middle cingulate cortex when individuals with ASD were asked to attribute actions to themselves, indicating a deficit in self-monitoring during interaction. Lombardo et al. (2009) similarly reported reduced functional connectivity between the ventromedial prefrontal cortex and somatosensory cortices—regions involved in self-other mapping. existential process usage, then, offers a linguistic marker of the developmental plateauing of the interpersonal self.

#### 4.5.5 Agency, body schema, and linguistic realization

The linguistic patterns observed in our data can also be interpreted in relation to the embodied sense of agency. Agency entails more than motor control; it involves recognizing oneself as the initiator of action in a socially meaningful context. In individuals with ASD, the subjective experience of agency is often less differentiated, particularly in interpersonal or affectively complex situations (Williams and Happé, 2009). This is not merely a behavioral matter but grounded in the neurophysiological organization of self-related cognition. Existential processes provide a linguistic alternative to transitive or volitional constructions. By focusing on what exists or is located, without marking an agent or initiator, existential processes enable speakers to describe the world while avoiding grammatical commitments to causality or volition. This strategy may appeal to speakers who experience uncertainty in attributing or interpreting actions with respect to themselves or others. These tendencies mirror known disruptions in body schema and sensorimotor awareness in ASD. Neuroimaging studies reveal atypical activation in the right temporoparietal junction and posterior parietal cortex—areas essential for spatial integration and proprioception (Devinsky, 2000; Mesulam, 2000; Tsakiris et al., 2008). Farrer et al. (2003) showed these same regions are critical for distinguishing self-generated from externally caused actions. The avoidance of agency in existential process constructions may thus reflect broader disruptions in embodied experience. Our data suggest that existential processes function not only as existential statements but also as compensatory forms that reduce the representational burden of interpersonal causality (Kato, 2024b).

#### 4.5.6 Inner speech, pronouns, and the self in language

Existential processes must also be considered within the broader context of self-referential language development. Atypical pronoun use—including pronoun reversal and third-person self-reference—is well documented in ASD (Kanner, 1943; Hobson, 1990; Tager-Flusberg, 1996), and reflects difficulties in adopting the deictic perspective needed to anchor the self in discourse (Kircher and David, 2003). These issues extend into the domain of inner speech. Individuals with ASD often rely less on internal verbalization and more on visual-spatial or motoric representations (Hurlburt et al., 1994; Whitehouse et al., 2006). Kennedy et al. (2006) found they report fewer autobiographical daydreams involving self or others, reinforcing the hypothesis that internal narrative frameworks—crucial for perspective-taking—are underdeveloped or qualitatively distinct. Kana et al. (2006) also report reduced connectivity between language and visual areas in ASD, consistent with less integrated verbal processing. existential processes, in this light, represent a linguistic manifestation of this cognitive profile. They allow reference without explicit speaker positioning. While consistent with Japanese norms of ellipsis and subject suppression (Ikegami, 2006), the frequency and distribution of existential processes in ASD speakers go beyond stylistic choice. As in Mach’s (1971) self-portrait—where the visual field is intact but the observer is absent— existential processes grammatically preserve the structure of perception while minimizing perspectival anchoring. They encode a mode of cognition that favors stability and perceptual focus over interpersonal dynamism.

In sum, ASD cognition is marked by a strong reliance on ecological self-awareness, with reduced integration of social and relational perspectives. This is linguistically reflected in a preference for existential processes, which emphasize existence over interaction (Kato, 2024b).

### 4.6 Repetitive and restricted behaviors (RRBs) discriminator: parallel clauses and LSR/expansion-elaboration-exemplifying

ASD individuals use parallel clauses significantly more, making them a discriminator in RRBs and cognitive rigidity. In LSR terms, this usage falls under *exemplifying*. The significantly higher use of parallel clauses in individuals with ASD reflects underlying cognitive differences that shape their linguistic structures and communication patterns. These structures allow speakers to list multiple events or actions in sequence, favoring explicit enumeration over conceptual integration. This pattern aligns with key cognitive characteristics of ASD, such as RRBs, WCC, executive function deficits, and predictive processing impairments. Through these mechanisms, ASD individuals develop a distinct linguistic style that prioritizes rigid, detail-focused, and repetitive speech patterns, making parallel clauses a prominent feature in their language use.

#### 4.6.1 Repetitive Speech and RRBs in ASD

ASD, as defined in the DSM-5 (American Psychiatric Association, 2013), is characterized by RRBs, which appear in both motor and linguistic patterns. These include motor stereotypies (e.g., hand-flapping, rocking), repetitive object use (e.g., lining up toys), and linguistic repetition (e.g., echolalia, rigid phrase repetition). In sentence structure, ASD individuals often list multiple examples in fixed syntactic patterns, reinforcing speech predictability and reducing cognitive effort. Parallel clauses function as a linguistic form of RRBs, promoting structured repetition. Instead of integrating ideas into a single cohesive thought, ASD individuals list actions sequentially, reflecting behavioral rigidity, where repetition provides structure and minimizes the need for linguistic flexibility. The following compares ASD and TD speakers:

(30) ASD speaker: *asa-ni okirarenakat-tari chūya gyakuten shi-tari.*
morning-LOC wake.up-NEG-PST-CONJ.REP a.bit woke.up-but soon sleep.again-CONJ
(Sometimes I couldn’t wake up in the morning, sometimes my sleep schedule was reversed, etc.).

(31) TD speaker: *asa okirarenai-koto-ga ōku chūya gyakutenshi-gachi-desu.*
morning wake.up-NEG-NMLZ-NOM often middle.school-GEN time a.little boring COP.PST
(I often can’t wake up in the morning and tend to have a reversed sleep schedule).

While the TD speaker generalizes, the ASD speaker lists experiences explicitly, avoiding abstraction and reinforcing repetitive linguistic patterns. This structured listing reflects perseveration, where monotropic attention makes shifting focus or reframing information difficult (Murray et al., 2005). It may also serve as a cognitive strategy to manage attention, helping ASD individuals maintain discourse fluency without frequent reorganization.

#### 4.6.2 WCC and detail-focused speech

WCC theory (Frith, 1989; Happé and Frith, 2006) suggests that individuals with ASD exhibit a strong preference for local detail processing rather than global meaning integration. Unlike TD individuals who synthesize multiple pieces of information into a broader, cohesive whole, ASD individuals focus on each component individually, treating them as discrete and independent entities rather than interrelated concepts.

This cognitive bias is directly reflected in language processing. While TD speakers often compress multiple ideas into a single summarized statement, ASD individuals list each detail explicitly, avoiding higher-level abstraction. ex., in describing weekend activities:

(32) TD Speaker: *shūmatsu-wa nonbirishite-ima-shita*.
weekend-TOP leisurely do-PROG.POL
(I had a relaxing weekend).

(33) ASD Speaker: *doyō-ni hon-yondari nichiyō-ni eiga-wo mitari-shimashita.*
Saturday-DAT book-read-REP, Sunday-DAT TOP cooking do-REP
(On Saturday, I read a book, and on Sunday, I watched a movie).

The TD speaker abstracts experiences into a single category (e.g., relaxing), while the ASD speaker lists events individually, reflecting difficulty in synthesizing details into a broader theme. ASD speech prioritizes enumeration over integration, reinforcing structured repetition.

ASD individuals also struggle with implicit meaning, leading them to explicitly state details rather than rely on listener inference. This lack of inferential processing supports their use of parallel clauses, allowing sequential presentation of details without requiring abstraction or interpretation.

#### 4.6.3 Executive function deficits and perseverative speech in ASD

Executive function deficits, particularly in cognitive flexibility and higher-order reasoning, are frequently observed in ASD (Lopez et al., 2005). These deficits contribute to rigid speech structures, leading ASD individuals to rely on familiar syntactic patterns, such as parallel clauses, which provide a stable linguistic framework.

(i) Cognitive rigidity and difficulty shifting discourse

ASD individuals struggle with shifting discourse patterns, leading to perseverative speech. Instead of summarizing efficiently, they list details repetitively within a fixed syntactic structure (e.g., *tari, toka, ka* constructions). Once established, they persist in using these patterns rather than adapting to new contexts, reinforcing rigid and repetitive speech. ex.: describing their interests:

(34) TD speaker: *eiga-ya dokusho ongaku-kanshō-ga shumi-desu.*
movies-AND reading music-appreciation-ETC do-PROG.POL
(My hobbies are movies, reading, and music appreciation).

(35) ASD speaker: *eiga-wo mi-tari hon-wo yondari ongaku-wo kii-tari-suru-no-ga suki-desu*
movie-ACC watch-REP, book-ACC read-REP do-PROG.POL
(I like watching movies, reading books, and listening to music).

The ASD speaker’s reliance on enumeration over categorization reinforces perseverative speech and reflects rigid cognitive processing.

(ii) Inhibitory control deficits and overproduction of examples

ASD individuals struggle to suppress unnecessary details, leading to verbose, repetitive speech even after conveying the main idea. ex., when asked about favorite foods:

(36) TD speaker: *karei-ya hanb*ā*gu-ga suki-desu*.
curry-NEX hamburger-NOM like-COP.POL
(I like curry and hamburgers).

(37) ASD speaker: *karei-ga suki-de hanb*ā*gu-mo suki-de r*ā*men-toka-mo suki-desu*.
curry-NOM like-CONJ hamburger-also like-COP.POL
(I like curry, hamburgers, and also ramen).

The ASD speaker’s continued listing reflects inhibition difficulties, making it hard to stop providing examples. This reinforces their reliance on parallel clauses as a structured, repetitive linguistic tool.

(iii) Predictive processing deficits and speech patterns in ASD

From the predictive processing theory (Van de Cruys et al., 2014) perspective, ASD individuals struggle with anticipating conversational information. This study suggests that prediction difficulties lead to overly explicit, detail-focused speech. Instead of filtering redundancy, they rely on bottom-up processing, making speech excessively precise or fragmented. Reduced sensitivity to implicit meaning and conversational cues further reinforces a rigid, sequential discourse style.

(38) TD speaker: *maiasa kimatta Ruthin-ga ari-masu*.
every.morning fix-PST routine-NOM exist.POL
(I have a set routine every morning).

(39) ASD speaker: *asa oki-te kao-wo arat-te go-han-wo tabe-te gakkō-ni it-te*
morning wake.up-CONJ face-ACC wash-CONJ school-LOC go.POL
(I wake up, wash my face, eat breakfast, go to school…).

Rather than anticipating the listener’s inference, the ASD speaker lists each detail explicitly, favoring enumeration over summarization.

In summary, ASD speech patterns reflect RRBs, marked by structured listing, perseveration, and reduced syntactic flexibility. Their reliance on parallel clauses, excessive examples, and difficulty shifting discourse indicates cognitive rigidity, inhibitory control deficits, and predictive processing challenges. Instead of integrating information fluidly, they prioritize explicit enumeration, resulting in overly detailed, fragmented, and inflexible speech.

### 4.7 Limitations and future perspectives

A key limitation of this study is the sample size, which affects the statistical robustness of the identified lexicogrammatical discriminators. Expanding the participant pool in future research will improve validation and reliability, strengthening the cognitive interpretation of these findings.

## 5 Conclusion

The findings of this study contribute to understanding how linguistic patterns relate to cognitive processing differences across the autism spectrum, and how lexicogrammatical tendencies manifest across the speech-language continuum. The position of an individual on the spectrum affects the degree to which these discriminators are present, with variations in how prominently they appear based on where the individual falls along the continuum of verbal ability. This study provides mechanistic insights for language intervention, offering clear indicators of what should be reinforced and what should be suppressed in language development. By understanding these linguistic tendencies in relation to cognitive traits, interventions can be tailored to the specific needs of individuals across the spectrum, addressing the full range of communicative abilities from minimally verbal to highly verbal individuals.

Building on these findings, it is important to recognize that each language has its own lexicogrammatical and pragmatic framework: ASD-related linguistic features vary across languages. Some lexicogrammatical discriminators appear in one language but not in another as seen in Japanese negotiating particles with no direct equivalents in English. This underscores the need for cross-linguistic studies to identify typological patterns in ASD-related language use. Multilingual corpora will help analyze ASD-related lexicogrammatical choices across languages, advancing research on neurocognitive variation.

## Data Availability

The original contributions presented in the study are included in the article/Supplementary material, further inquiries can be directed to the corresponding author.
