# Supplementary material for: Cognitive constraints and lexicogrammatical variability in ASD: from diagnostic discriminators to intervention strategies
Source: Front Hum Neurosci. 2025 Aug 1;19:1606701. doi: 10.3389/fnhum.2025.1606701 (PMC12366435; doi:10.3389/fnhum.2025.1606701)
Supplement: Supplementary file 1 [file Data_Sheet_1.docx]

**Supplementary Material**

**Table S1**. Overview of Demographic and Clinical Metrics in ASD vs. Non-ASD Populations.

| Characteristic | ASD Group | Non-ASD Group | P-Value |
| --- | --- | --- | --- |
|  | (N=64) | (N=71) |  |
| Age (years) | 18 ± 3.48 | 19 ± 2.77 | 0 |
| Sex (M/F) | 24/40 | 39/32 | 0.06 |
| Education | N/A | College GPA range: 2.4 - 2.8 | N/A |
| ADOS-2 Module 3 | 6.93 ± 1.38 | 2.75 ± 2.01 | < 0.01 |
| ADOS-2 Module 4 | 11.42 ± 3.55 | 4.22 ± 2.17 | < 0.01 |
| SRS-2 Total Score | 85.53 ± 9.00 | N/A | N/A |
| WISC-IV-IQ Full Scale IQ | 81.22 ± 14.42 | N/A | N/A |
| WAIS-III Full Scale IQ | 91.33 ± 20.12 | N/A | N/A |
| Vineland-II Composite | 64.83 ± 22.53 | N/A | N/A |
| AQ | 36.64 ± 8.04 | N/A | N/A |
| PARS-TR Preschoolers | 13.54 ± 6.27 | N/A | N/A |
| PARS-TR Adolescents & adults | 24.30 ± 11.49 | N/A | N/A |

**Table S2.** Tag types and linguistic functions.

| **Lexicogrammar headings** | **Linguistic functions** | | **Tag types** | **No. of tag types** |
| --- | --- | --- | --- | --- |
| **Ideational metafunction** | | | | |
| 1. Process type | The mental image of reality is constructed by the TRANSITIVITY (clause component) of a clause. All individuals create a representation of reality. Experiential worlds are defined using 10 types of process verbs, yielding information about how, when speaking, an individual creates a representation of reality. | | 1.Material-doing 2.Material-happen 3.Mental-cognition 4.Mental-affect 5.Mental-perception 6.Relational-attribute 7.Relational-identity 8.Behavioral 9.Verbal 10.Existential | 10 |
| 2. Ergativity | This measures causation or instigation. In an ergative analysis, the participant that causes an event is the agent. Ergativity reveals whether a speaker interprets events and reality from the causal viewpoint of agency (effective) or becoming (i.e., a perspective lacking agency; a middle). | | 1.effective 2.middle | 2 |
| 3. Transitivity | A property that yields clues regarding the perspective (active or passive) from which the speaker interprets events and reality. | | voice (1.passive/active 2.causative) | 2 |
| 4. Clause complexes | The Japanese sentence type (of 22 types) chosen. This reveals syntactic ability and any cognitive tendency or deficiency. | | 1.Parallel clauses 2.*Te*-form/Conjunctive clauses-parallel/contrast 3.*Te*-form/Conjunctive clauses- forerunner 4.*Te*-form/Conjunctive clauses-sequence of actions 5.*Te*-form/Conjunctive clauses-cause/ reason 6.*Te*-form/Conjunctive clauses-adversative connective 7.*Te*-form/Conjunctive clauses-resultative condition 8.*Te*-form/Conjunctive clauses-attendant circumstance 9.Conditional clauses-resultative condition 10.Conditional clauses-converse condition-converse condition 11.Conditional clauses-converse condition-adversative connective 12.Conditional clauses-cause/ reason 13.Purpose clauses 14.Time clauses-temporal anteroposterior relation 15.Time clauses-simultaneous actions 16.Time clauses-others 17.Manner clauses 18.Reported clauses 19.Interrogative clauses 20.Noun clauses 21.Adnominal clauses 22.Cordinate clauses | 22 |
| 5. Logico- semantic relation | Logical clause linkages revealing syntactic ability, discourse strategy, and any cognitive tendency or deficiency. | | 1.Expansion-elaboration-expository 2.Expansion-elaboration-exemplifying 3.Expansion-elaboration-clarifying 4.Expansion-extension-additive 5.Expansion-extension-alternative 6.Expansion-enhancement-temporal 7.Expansion-enhancement-spatial 8.Expansion-enhancement-manner 9.Expansion-enhancement-cause-conditional 10.Projection-quote 11.Projection-report 12.Projection-idea 13.Projection-embedding | 13 |
| 6. Auxiliary verbs | **Stative:** Verbs describing the state of a subject rather than an action, reflecting the perspective of a speaker on an ongoing phenomenon. | | stative: (9 categories) compound: (1 category) | 10 |
|  | **Compound:** Verbs created by adding one verb to the stem of another; use of these verbs reflects the morphological skill of a speaker. | |  |  |
| **Interpersonal metafunction** | | | | |
| 7. Modality | In SFL, modality refers to an area of meaning that lies between yes and no; this constitutes the intermediate space between positive and negative polarity, categorized as either modalization (epistemic modality) and modulation. | | 1.Ability 2.Probability 3.Usuality 4.Necessity 5.Obligation 6.Permission 7.Expectation 8.Inclination 9.Modal Adjunct/Probability 10.Modal Adjunct/Usuality | 10 |
| 8. Appraisal- attitude | The semantic resource used to negotiate emotional reactions, judge behavior, and value things. Attitude is divided into three domains: affect, judgment, and appreciation. Affect is used to interpret emotional responses (including fear, loathing, sadness, and happiness); judgment is used for moral evaluation of behavior (including ethical, brave, and deceptive); and appreciation is used to interpret the esthetic qualities of semiotic phrases/processes and natural phenomena (including remarkable, desirable, elegant, harmonious, and innovative). This lexicogrammar reveals the speaker’s value system. | | 1.AFFECT-inclination 2.AFFECT-emotion 3.AFFECT- security 4.AFFECT-satisfaction 5.JUDGEMENT-capacity 6. JUDGEMENT-reliability 7.JUDGEMENT-veracity 8.JUDGEMENT-propriety 9.JUDGEMENT-propencity 10.APPRECIATION-reaction 11.APPRECIATION-composition 12.APPRECIATION-phase-time 13.APPRECIATION-phase-extent 14.APPRECIATION-phase-degree 15.APPRECIATION-phase-space 16.APPRECIATION-phase-distance 17.APPRECIATION-phase-mass 18.APPRECIATION-social evaluation | 18 |
| 9. Appraisal- graduation | This is one of the three categories that make up Appraisal, along with Appraisal-attitude, which focuses on gradability. (i.e., adjustment of the extent of evaluation). | | 1.FORCE-intensification 2.FORCE-quantification 3.FOCUS-sharpening 4.FOCUS-softening | 4 |
| 10. Negotiating particles | A lexis that adds various negotiatory values to a clause, implying the attitudinal stance of a speaker toward a proposition or proposal; this lexis is associated with a call for attention and indicates the territory of the information involved. | | sentence-final:1.*kana* 2.*kane* 3.*sa* 4.*ne* 5.*yo* 6.*yona* 7.*yone* Mid sentence:8.*kane* 9.*sa* 10.*ne* 11.*yo* Other:12.*sa* 13.*ne* | 13 |
| 11. Explanatory mood | An optional lexicogrammar often added to other mood types such as declarative and interrogative, implying a variety of meanings. It constitutes a cause, reason, motivation, source, and/or grounds for judgment that suggest a causal relationship between the explained and the explainer. | | 1.Explanatory mood 2.Explanatory mood:*ka* 3.Explanatory mood:*kana* 4.Explanatory mood:*kane* 5.Explanatory mood:*kedo* 6.Explanatory mood:other 7.Explanatory mood:*na* 8.Explanatory mood:*ne* 9.Explanatory mood:*yo* 10.Explanatory mood:*yone* 11.Explanatory mood:*yona* 12.Explanatory mood:*monoda* | 12 |
| 12. Evidentiality | This lexicogrammar describes how a speaker judges the validity of a proposition. Three types of evidence are used. *Appearance* refers to how the information is likely to appear or eventually occur; *hearsay* refers to how it will be known whether the event occurs; and *reasoning* refers to the reason the judgment is made or how the event is known to happen. | | 1.appearance 2.hearsay 3.reasoning | 3 |
| 13. Optative mood | A *desire* or *urge* to do something that the speaker considers desirable. | | lexis to express desire to do something | 1 |
| 14. Auxiliary verbs, benefactive | Verbs used when two parties converse; one party is doing something that benefits the other, and the other party is the recipient of that benefit. Such verbs indicate whether the speaker positions the other party inside or outside. | | Benefactive: (10 categories) | 10 |
| 15. Onomatopoeia | Imitative and mimetic words used to express manner, quality, or an exclamation. | | 1.imitative words 2.imitative mimetic words | 2 |
| 16. Filler | A time-filler: a meaningless sound, word, or phrase used in social settings when an individual is aware that a listener is present. | | Filler words-1.*maa* 2.*nanka* 3.*ano* 4.*unto* 5.*eeto* 6.*sono* 7.*kono* 8.*kou* | 8 |
|  |  |  | Total | 140 |

**Table S3.** Statistical Significance of Lexicogrammatical Discriminators in Differentiating ASD from Non-ASD Lexicogrammatical Choices.

| Lexicogrammar | Mean | SD | p-value |
| --- | --- | --- | --- |
| Auxiliary Verbs-Benefactive do (for someone) | -0.0052 | 0.0018 | 0.0031 |
| Auxiliary Verbs-Stative-do (end up / ended up, implying regret/vexation) | -0.0183 | 0.0075 | 0.0148 |
| Auxiliary Verbs-Stative-try (doing/to do something and observe outcome) | -0.0070 | 0.0032 | 0.0256 |
| Clause complexes-Noun clauses | -0.0503 | 0.0201 | 0.0124 |
| Clause complexes-Reported clauses | -0.0741 | 0.0214 | 0.0005 |
| Clause complexes-Adnominal clauses | -0.0512 | 0.0177 | 0.0038 |
| Clause complexes/*Te-*form Conjunctive clauses (parallel/contrast) | -0.0247 | 0.0069 | 0.0004 |
| Clause complexes/*Te-*form/Conjunctive clauses-forerunner | -0.0074 | 0.0030 | 0.0136 |
| Clause complexes/*Te-*form/Conjunctive clauses-cause/reason | -0.0411 | 0.0103 | 0.0001 |
| Clause complexes/*Te-*form/Conjunctive clauses-attendant circumstance | -0.0185 | 0.0079 | 0.0181 |
| Clause complexes/*Te-*form/Conjunctive clauses-sequence of actions | -0.0223 | 0.0084 | 0.0079 |
| Clause complexes-Parallel clauses | 0.0808 | 0.0173 | 0.0000 |
| Clause complexes-Conditional clauses-cause/reason | -0.0381 | 0.0151 | 0.0114 |
| Clause complexes-Conditional clauses-resultative condition | -0.0227 | 0.0111 | 0.0410 |
| Logico-Semantic Relation/Projection-embedding | -0.1052 | 0.0286 | 0.0002 |
| Logico-Semantic Relation/Projection-idea | -0.0683 | 0.0191 | 0.0003 |
| Logico-Semantic Relation/Expansion-enhancement-manner | -0.0196 | 0.0083 | 0.0181 |
| Logico-Semantic Relation/Expansion-enhancement-cause-conditional | -0.1070 | 0.0250 | 0.0000 |
| Logico-Semantic Relation/Expansion-extension-additive | -0.0860 | 0.0221 | 0.0001 |
| Logico-Semantic Relation/Expansion-elaboration-exemplifying | 0.0833 | 0.0172 | 0.0000 |
| Process Type/Existential | 0.1336 | 0.0292 | 0.0000 |
| Process Type/Relational-attribute | -0.0849 | 0.0316 | 0.0072 |
| Appraisal/Attitude/JUDGEMENT-propriety | -0.0096 | 0.0042 | 0.0235 |
| Appraisal/Attitude/JUDGEMENT-veracity | -0.0124 | 0.0052 | 0.0163 |
| Appraisal/Attitude/AFFECT-satisfaction | -0.0328 | 0.0049 | 0.0000 |
| Appraisal/Attitude/APPRECIATION-phase-space | -0.0024 | 0.0011 | 0.0236 |
| Appraisal/Attitude/APPRECIATION-reaction | -0.0533 | 0.0206 | 0.0098 |
| Appraisal/Graduation/FORCE-intensification | -0.1595 | 0.0210 | 0.0000 |
| Appraisal/Graduation/FORCE-quantification | -0.0122 | 0.0057 | 0.0333 |
| Evidentiality/Appearance | -0.0094 | 0.0041 | 0.0225 |
| Modality/Modal Adjunct/Probability | -0.0201 | 0.0064 | 0.0016 |
| Modality/Modalization/Probability | -0.0345 | 0.0169 | 0.0410 |
| Modality/Modulation/Obligation | -0.0042 | 0.0015 | 0.0047 |
| Negotiating particles-sentence-final:*kane* | -0.0334 | 0.0103 | 0.0012 |
| Negotiating particles-sentence-final:*ne* | -0.1290 | 0.0281 | 0.0000 |
| Negotiating particles-sentence-final: *yo* | -0.0329 | 0.0081 | 0.0000 |
| Negotiating particles-sentence-final: *yone* | -0.0146 | 0.0042 | 0.0005 |
| Negotiating particle-Mid sentence:*kane* | -0.0023 | 0.0009 | 0.0072 |
| Negotiating particles-Mid sentence:*ne* | -0.0363 | 0.0102 | 0.0004 |
| Mood/Explanatory mood:*kedo* | -0.0347 | 0.0137 | 0.0111 |
| Mood/Explanatory mood:*ne* | -0.0022 | 0.0010 | 0.0199 |
| Mood/Explanatory mood:*yo* | -0.0259 | 0.0063 | 0.0000 |
| Mood/Explanatrory mood:*yone* | -0.0065 | 0.0022 | 0.0041 |
| Filler/*unto* | 0.0354 | 0.0135 | 0.0088 |
| Filler/*kono* | -0.0017 | 0.0005 | 0.0021 |
| Onomatopoeia/imitative mimetic words | -0.0126 | 0.0059 | 0.0315 |
| Ergativity/Middle | 0.0050 | 0.0774 | 0.9481 |
| Ergativity/Effective | -0.0528 | 0.0357 | 0.1396 |
| Auxiliary Verbs/Benefactive-have someone(superior)do something for me / my group | -0.0003 | 0.0003 | 0.3146 |
| Auxiliary Verbs/Benefactive-superior does for me / my group | 0.0005 | 0.0013 | 0.7013 |
| Auxiliary Verbs/Benefactive-someone does for me / my group | 0.0064 | 0.0051 | 0.2124 |
| Auxiliary Verbs/Benefactive-have someone do something for me / my group | -0.0041 | 0.0027 | 0.1300 |
| Auxiliary Verbs/Benefactive-do～for someone (inferior) | -0.0003 | 0.0003 | 0.3152 |
| Auxiliary Verbs/Benefactive-do～(command or instruction) | 0.0001 | 0.0005 | 0.8442 |
| Auxiliary Verbs/stative-is;are(a state resulting from the action) | 0.0003 | 0.0004 | 0.4672 |
| Auxiliary Verbs/stative-manner at time of movement/to;into/doing something and then going/will continue/disappearance of something or becoming more distant from the speaker | 0.0028 | 0.0035 | 0.4262 |
| Auxiliary Verbs/stative-is ～ing(duration) / has been; is (result) / always (repetition) / narrative present tense(experience) / -s, ～ing(state) | 0.0067 | 0.0193 | 0.7284 |
| Auxiliary Verbs/stative-do something for a future or on a temporary basis / leave a state for a future purpose | -0.0023 | 0.0016 | 0.1430 |
| Auxiliary Verbs/stative-manner at time of movement / to;into(movement toward something or someone) / doing something and then comeing / have～ (duration of an action or a change) / start to～（emergence, inception) / come to(action directed toward speaker) | -0.0045 | 0.0055 | 0.4159 |
| Auxiliary Verbs/compound | -0.0110 | 0.0075 | 0.1430 |
| Clause complexes/Cordinate clauses | -0.0200 | 0.0148 | 0.1774 |
| Clause complexes/Interrogative clauses | -0.0037 | 0.0036 | 0.3018 |
| Clause complexes/*Te*-form/Conjunctive clauses-adversative connective | -0.0020 | 0.0011 | 0.0676 |
| Clause complexes/*Te*-form/Conjunctive clauses-resultative condition | -0.0012 | 0.0015 | 0.4289 |
| Clause complexes/Time clauses-others | 0.0007 | 0.0014 | 0.6088 |
| Clause complexes/Time clauses-temporal anteroposterior relation | -0.0041 | 0.0036 | 0.2558 |
| Clause complexes/Time clauses-simultaneous actions | -0.0027 | 0.0055 | 0.6311 |
| Clause complexes/Conditional clauses-converse condition-adversative connective | -0.0076 | 0.0039 | 0.0532 |
| Clause complexes/Conditional clauses-converse condition-converse condition | -0.0016 | 0.0036 | 0.6656 |
| Clause complexes/Manner clauses | -0.0014 | 0.0029 | 0.6342 |
| Clause complexes/Purpose clauses | -0.0036 | 0.0031 | 0.2520 |
| Logico-Semantic Relation/Projection-report | 0.0027 | 0.0021 | 0.2009 |
| Logico-Semantic Relation/Projection-quote | -0.0095 | 0.0118 | 0.4194 |
| Logico-Semantic Relation/Expansion-enhancement-temporal | -0.0060 | 0.0073 | 0.4124 |
| Logico-Semantic Relation/Expansion-enhancement-spatial | -0.0005 | 0.0003 | 0.1271 |
| Logico-Semantic Relation/Expansion-extention-alternative | -0.0024 | 0.0023 | 0.2985 |
| Logico-Semantic Relation/Expansion-elaboration-expository | 0.0022 | 0.0014 | 0.1078 |
| Logico-Semantic Relation/Expansion-elaboration-clarifying | -0.0012 | 0.0036 | 0.7410 |
| Process Type/Mental-affect | -0.0392 | 0.0248 | 0.1147 |
| Process Type/Mental-perception | -0.0035 | 0.0040 | 0.3868 |
| Process Type/Mental-cognition | 0.0118 | 0.0324 | 0.7144 |
| Process Type/Material-doing | -0.0352 | 0.0343 | 0.3047 |
| Process Type/Material-happen | 0.0026 | 0.0099 | 0.7927 |
| Process Type/Verbal | -0.0082 | 0.0176 | 0.6400 |
| Process Type/Behavioral | -0.0028 | 0.0102 | 0.7857 |
| Process Type/Relational-identity | -0.0208 | 0.0265 | 0.4319 |
| Voice/passive/active | 0.0034 | 0.0034 | 0.3136 |
| Voice/causative | -0.0059 | 0.0092 | 0.5200 |
| Appraisal/Attitude/JUDGEMENT-reliability | -0.0019 | 0.0030 | 0.5264 |
| Appraisal/Attitude/JUDGEMENT-propencity | -0.0277 | 0.0147 | 0.0588 |
| Appraisal/Attitude/JUDGEMENT-capacity | -0.0088 | 0.0065 | 0.1769 |
| Appraisal/Attitude/AFFECT-security | 0.0180 | 0.0134 | 0.1778 |
| Appraisal/Attitude/AFFECT-emotion | -0.0090 | 0.0119 | 0.4516 |
| Appraisal/Attitude/AFFECT-inclination | -0.0220 | 0.0127 | 0.0838 |
| Appraisal/Attitude/APPRECIATION-phase-time | -0.0011 | 0.0052 | 0.8348 |
| Appraisal/Attitude/APPRECIATION-phase-degree | -0.0036 | 0.0041 | 0.3797 |
| Appraisal/Attitude/APPRECIATION-phase-extent | 0.0007 | 0.0050 | 0.8929 |
| Appraisal/Attitude/APPRECIATION-phase-distance | 0.0001 | 0.0053 | 0.9818 |
| Appraisal/Attitude/APPRECIATION-phase-mass | 0.0000 | 0.0052 | 0.9929 |
| Appraisal/Attitude/APPRECIATION-composition | 0.0013 | 0.0099 | 0.8993 |
| Appraisal/Attitude/APPRECIATION-social evaluation | 0.0004 | 0.0012 | 0.7315 |
| Appraisal/Graduation/FORCE-sharpening | -0.0011 | 0.0133 | 0.9353 |
| Appraisal/Graduation/FOCUS-softening | -0.0188 | 0.0252 | 0.4550 |
| Evidentiality/hearsay | 0.0005 | 0.0005 | 0.3196 |
| Evidentiality/reasoning | 0.0011 | 0.0020 | 0.5657 |
| Modality/Modal Adjunct/Usuality | 0.0093 | 0.0072 | 0.1974 |
| Modality/Modalization/Ability | 0.0049 | 0.0092 | 0.5919 |
| Modality/Modalization/Usuality | 0.0070 | 0.0049 | 0.1542 |
| Modality/Modulation/Necessity | -0.0029 | 0.0045 | 0.5164 |
| Modality/Modulation/Inclination | 0.0022 | 0.0018 | 0.2286 |
| Modality/Modulation/Expectation | -0.0001 | 0.0016 | 0.9739 |
| Modality/Modulation/Permission | -0.0022 | 0.0015 | 0.1435 |
| Negotiator particles-other:*sa* | 0.0004 | 0.0006 | 0.5453 |
| Negotiator particles-other:*ne* | -0.0001 | 0.0003 | 0.7624 |
| Negotiator particles-sentence-final:*kana* | 0.0117 | 0.0123 | 0.3424 |
| Negotiator particles-sentence-final:*sa* | 0.0006 | 0.0004 | 0.1018 |
| Negotiator particles-sentence-final:*yona* | -0.0002 | 0.0007 | 0.7599 |
| Negotiator particle-Mid sentence:*sa* | 0.0022 | 0.0015 | 0.1241 |
| Negotiator particle-Mid sentence:*yo* | -0.0002 | 0.0001 | 0.1607 |
| Mood/Explanatory mood | 0.0050 | 0.0040 | 0.2155 |
| Mood/Explanatory mood:*ka* | -0.0066 | 0.0035 | 0.0559 |
| Mood/Explanatory mood:*kana* | 0.0051 | 0.0060 | 0.3954 |
| Mood/Explanatory mood:*kane* | -0.0032 | 0.0020 | 0.1166 |
| Mood/Explanatory mood:other | 0.0023 | 0.0054 | 0.6718 |
| Mood/Explanatory mood:*na* | 0.0009 | 0.0013 | 0.5132 |
| Mood/Explanatory mood:*yona* | 0.0003 | 0.0006 | 0.5386 |
| Mood/Explanatory mood:*monoda* | 0.0004 | 0.0010 | 0.6709 |
| Mood/Optative Mood/lexis to express desire to do something | -0.0068 | 0.0081 | 0.4000 |
| Filler/*ano* | 0.0540 | 0.0366 | 0.1398 |
| Filler/*eeto* | -0.0525 | 0.0390 | 0.1785 |
| Filler/*kou* | 0.0001 | 0.0316 | 0.9979 |
| Filler/*sono* | -0.0168 | 0.0112 | 0.1346 |
| Filler/*nanka* | -0.0412 | 0.0324 | 0.2034 |
| Filler/*maa* | 0.0102 | 0.0543 | 0.8514 |
| Onomatopoeia/imitative words | -0.0009 | 0.0025 | 0.7316 |

**Table S4.** Abbreviations used in interlinear glossing.

| Abbreviation | Full spelling | Notes |
| --- | --- | --- |
| ACC | accusative |  |
| APP | appearance | Use when emphasizing appearance (～*sō*) |
| BEN | benefactive | Use for ～*tekureru*, ～*temorau* etc. |
| COMP | complementizer | e.g., *to* in Japanese |
| COND | conditional |  |
| CONJ | conjunction | Use when specific type is not required |
| COORD | coordinator | For and/or-type coordination particles like *ya*, *to* |
| COP | copula |  |
| DAT | dative |  |
| ETC | et cetera | Used for Japanese *toka*, *nado*, meaning “and so on”, “for example” |
| EVD | evidential | For hearsay, inference, appearance forms like ～*sō* |
| FP | final particle | Sentence-final discourse marker |
| GEN | genitive |  |
| HEARSAY | hearsay evidential | Common glossing for the Japanese form 'sōda' when used as hearsay. |
| INF | inferential | For internal judgments (e.g., ～*darō*) |
| LOC | locative |  |
| MOD | modal | For epistemic/deontic modals |
| NEG | negative |  |
| NEX | non-exhaustive | For *ya, to* (non-exhaustive coordination/listing) |
| NMLZ | nominalizer |  |
| NOM | nominative |  |
| POL | polite | Appropriate for Japanese honorific forms |
| PROG | progressive |  |
| PST | past |  |
| Q | question particle |  |
| REP | representative conjunctive | used to gloss Japanese verbal ending “-tari” |
| TOP | topic | Common in Japanese glossing |


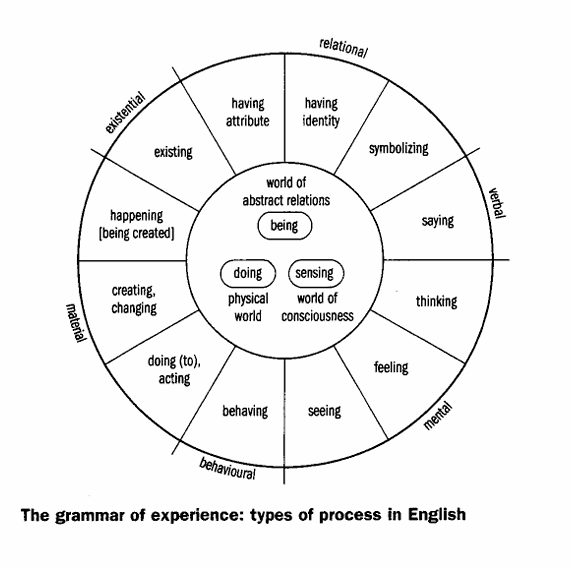


**Figure S1.** The grammar of experience: types of process in English.
